# Alleviation of endoplasmic reticulum stress protects against cisplatin-induced ovarian damage

**DOI:** 10.1186/s12958-018-0404-4

**Published:** 2018-09-03

**Authors:** Yuping Wu, Congshun Ma, Huihui Zhao, Yuxia Zhou, Zhenguo Chen, Liping Wang

**Affiliations:** 1 Department of Obstetrics and Gynecology, Nanfang Hospital, Southern Medical University, Guangzhou, 510515 China; 2 Reproductive Medicine Center, Guangdong Provincial Family Planning Special Hospital, Guangzhou, 510699 China; 3 Department of Cell Biology, School of Basic Medical Sciences, Southern Medical University, Guangzhou, 510515 China

**Keywords:** Cisplatin, Endoplasmic reticulum stress, Granulosa cell apoptosis, Ovarian damage, 4-PBA

## Abstract

**Background:**

Cisplatin (CDDP), a widely used chemotherapeutic agent, can induce excessive granulosa cell apoptosis, follicle loss and even premature ovarian insufficiency (POI). However, the mechanism remains elusive, although some studies have indicated the involvement of endoplasmic reticulum stress (ERS). The aim of our study was to investigate the possible mechanism ERS in CDDP-induced granulosa cell apoptosis and follicle loss.

**Methods:**

A POI mouse model was generated by CDDP. The ovaries samples were collected and processed for isobaric tags for relative and absolute quantification analysis (iTRAQ) to screen out our interested proteins of HSPA5 and HSP90AB1, and the decline in their expression were verified by a real-time quantitative PCR and a western blotting assay. In vitro, human granulosa cells, KGN and COV434 cells were transfected with siRNA targeting *HSPA5* and *HSP90AB1* and then treated with CDDP, or treated with CDDP with/without CDDP+ 4-phenylbutyric acid (4-PBA) and 3-methyladenine (3-MA). The levels of ERS, autophagy and apoptosis were evaluated by western blotting, DALGreen staining and flow cytometry. In vivo, ovaries from mice that received intraperitoneal injections of saline, CDDP, CDDP+ 4-PBA and CDDP+ 3-MA were assayed by immunofluorescence, hematoxylin and eosin (H&E) staining for follicle counting, and terminal-deoxynucleotidyltransferase-mediated dUTP nick end labeling (TUNEL) staining for cell apoptosis assay. The plasma hormone levels were measured by an enzyme-linked immunosorbent assay (ELISA) kit.

**Results:**

We have clarified the relationships between ERS, autophagy, and apoptosis in CDDP-induced granulosa cell apoptosis, both in vitro and in vivo. Alleviating ERS by inhibiting HSPA5 and HSP90AB1 attenuated CDDP-induced autophagy and apoptosis. 4-PBA treatment significantly attenuated CDDP-induced cell autophagy and apoptosis in cultured KGN and COV434 cells. However, inhibiting cell autophagy with 3-MA negligibly restored the CDDP-induced changes in ERS and apoptosis. In vivo experiments also demonstrated that treatment with 4-PBA, but not 3-MA, prevented CDDP-induced ovarian damage and hormone dysregulation.

**Conclusions:**

CDDP-induced ERS could promote autophagy and apoptosis in granulosa cells, causing excessive follicle loss and endocrine disorders. Alleviation of ERS with 4-PBA, but not of autophagy with 3-MA, protect against CDDP-induced granulosa cell apoptosis and ovarian damage. Thus, 4-PBA can be used to protect the ovary during chemotherapy in women.

**Electronic supplementary material:**

The online version of this article (10.1186/s12958-018-0404-4) contains supplementary material, which is available to authorized users.

## Background

Premature ovarian insufficiency (POI), previously called premature ovarian failure (POF), is described as the cessation of ovarian function before the age of 40 years [1]. The diagnosis is based on oligo- or amenorrhea for at least 4 months and an elevated follicle-stimulating hormone (FSH) level of > 25 IU/l on two occasions > 4 weeks apart [2]. The decline in ovarian functions is related to the loss of the resting follicles and the reduced biological competence of atresia follicles [3]. The orchestrated cross-talk between the oocytes and the surrounding granulosa cells is necessary for folliculogenesis [4]. Therefore, the loss of homeostasis in granulosa cells often leads to POI [5].

Chemotherapeutic treatments frequently causes ovarian damage [6]. Cisplatin (CDDP) is an anticancer agent widely used alone or in combination with other chemotherapeutic agents, and has been a key component of first-line chemotherapy against broad range of cancers [7]. However, nonselective distribution of the drug between normal and tumor tissue causes severe side effects [8]. CDDP can induce DNA damage and cell apoptosis, reduce follicle reserve and decrease steroidogenic activity, and even impairs female reproduction [9]. Previous studies have demonstrated various signaling pathways that participate in CDDP-induced apoptosis on granulosa cells. Several members of the Wnt signaling pathway were found to be downregulated in granulosa cells after exposure to CDDP, and restoration of β-catenin signaling protected granulosa cells from the injury induced by CDDP [10]. CDDP can also damage the DNA of granulosa cells by upregulation p63 and activated c-Abl-dependant pathway [11]. Additionally, p53 can induce granulosa cell apoptosis after CDDP treatment, by the transactivation of pro-apoptotic genes such as *Bax30*, *NOXA* and *PUMA,* or by relocating at the mitochondrion [12]. However, the detailed mechanisms underlying the ovarian damage caused by CDDP are still unclear.

After the discovery of the death receptor signaling and mitochondrial pathways, it was demonstrated that endoplasmic reticulum stress (ERS) can lead to apoptosis [13]. ERS occurs when mutant proteins disrupt protein folding in the ER, and ERS activates a signaling network called “the unfolded protein response” (UPR) [14]. Excessive and persistent ERS leads to cell dysfunction or even death [15, 16]. Recently, several studies have suggested that ERS promotes cell apoptosis and is related to follicular atresia, for which an ERS-mediated mechanism of cell autophagy and apoptosis has been proposed [16, 17]. In contrast, another study suggested that ERS inhibits autophagy [18]. Therefore, the exact effects of ERS on cell fate and its role in CDDP-induced ovarian damage remain to be clarified.

In this study, we generated a mouse model of POI with the intraperitoneal injection of CDDP for 7 days. The whole mouse ovaries were then subjected to proteomic screening using isobaric tags for relative and absolute quantification (iTRAQ) analysis. The results showed that two ERS-related proteins, 78-kDa glucose-regulated protein (HSPA5, GRP78, or BiP) and heat shock protein HSP90-beta (HSP90AB1, HSP84, or TSTA) were strongly associated with CDDP-induced ovarian damage. We then found that both of them were predominantly expressed in the granulosa cells from secondary and antral follicles. Thus, we hypothesize that HSPA5 and HSP90AB1 play key roles in CDDP-induced granulosa cell apoptosis and ovarian damage. Therefore, we designed in vitro and in vivo experiments using small interfering RNAs (siRNAs) directed against *HSPA5* and *HSP90AB1* and an inhibitor of ERS, 4-phenylbutyric acid (4-PBA), to clarify the roles of ERS in CDDP-induced cell autophagy, granulosa cell apoptosis and ovarian damage.

## Methods

### Animals

Six-week-old wild-type female C57BL/6 J mice were from the Southern Medical University Animal Center (Guangzhou, China). The mice were housed in a temperature- and humidity-controlled animal facility and maintained on a 12-h light/dark cycle. They were acclimated for 5 days before the experiment, with free access to a commercial rodent diet and tap water. All animal experiments were approved by the Southern Medical University Committee on the Use and Care of Animals and were performed in accordance with the Committee’s guidelines and regulations.

### POI model

Twenty mice were randomly and evenly divided into two groups. The experimental group received intraperitoneal injections of CDDP (2.5 mg/kg/d in saline) (Sigma-Aldrich, Shanghai, China) for 7 consecutive days, and the control group were injected with saline. This dosage was according to a previous study [19] and our preliminary results showing that this is the minimum dose causing significant morphological changes within 7 days when compared with the control group. After anesthesia induced with 10% chloral hydrate, the mice were killed and their ovaries rapidly dissected. Ovaries of three mice randomly selected from each group were subjected to a proteomic analysis with iTRAQ (Fitgene Biotech, Guangzhou, China). The remaining ovaries were used for real-time quantitative PCR (qPCR) and a western blotting analysis. Ovaries for histological examination were fixed in 4% formaldehyde.

### Protein preparation

The frozen ovaries were grinded by liquid nitrogen and then lysed in 500 μl of LC3 SDS lysis buffer (Add 1× PMSF before use) containing 7 M Urea, 2 M Thiourea, 20 mM Tris base and 0.2% SDS. Then the samples were sonicated and centrifuged to collect the supernatant. Every 250 μL of supernatant was precipitated overnight at − 20 °C with 1 mL acetone. After discarding the acetone and air drying, the resulting pellet was dissolved in lysate L3 (no SDS). Then the sample was sonicated on ice and centrifuged at 12,000×g for 20 min at 4 °C, the supernatant was collected. Protein concentration was estimated by the Bradford method (Fitgene Biotech, Guangzhou, China).

### Trypsin digestion and iTRAQ labeling

iTRAQ labeling was performed according to the manufacturer’s protocol (Applied Biosystems, Sciex). Briefly, 200 μg of each protein sample was reduced with TCEP Reducing Reagent at 60 °C for 1 h,and alkylated with MMTS Cysteine-Blocking Reagent at room temperature for 30 min. Then, proteins were digested with trypsin (Promega, USA) at 37 °C at a ratio of 1:50 (enzyme-to-substrate) overnight. Each sample was labeled separately with two of the eight available tags (control: 114 and 116 tags; cisplatin: 117 and 119 tags). All labeled peptides were pooled together.

### High-pH reversed-phase chromatography

The Ultimate 3000 HPLC system (Dionex, USA) equipped with a 2.00-mm-inner diameter *100-mm-long Gemini-NX 3u C18110Acolumns (Phenomenex, USA) was used for High-pH fractionation. Peptides were loaded onto the column and washed isocratically at 95% eluent A (20 mM HCOONH4, 2 M NaOH) (pH 10). The iTRAQ-tagged peptides fractionation was performed using a linear binary gradient from 15 to 50% B (20 mM HCOONH 4, 2 M NaOH, 80% ACN) (pH 10) at 0.2 ml/min for more than 45 min. Finally, the column was washed at 90% B for 10 min and returned to 95% A for 10 min. Set the UV detector was at 214/280 nm, and fractions were collected every 1 min. 10 fractions were pooled and dried by vacuum centrifuge for subsequent nano-reversed phase liquid chromatography (nano-LC) fractionation.

### RPLC-MS/MS analysis

The fraction was resuspended in loading buffer (0.1% FA,2% ACN) and separated with an Ultimate 3000 nano-LC system equipped with a C18 reverse phase column (100-μm inner diameter, 10-cm long, 3-μm resin from MichromBioresources, Auburn, CA). Separate the peptides with the following parameters: 1) mobile phase A:0.1% FA, 5% ACN, dissolved in water; 2) mobile phase B: 0.1% FA, 95% ACN; 3) flow rate: 300 nl/min; 4) gradient: B-phase increased from 5 to 40%,70 min. Then, the LC eluent was subject to Q Exactive (Thermo Fisher) in an information dependent acquisition mode. MS spectra were acquired across the mass range of 400–1250 m/z in high resolution mode (> 30,000) using 250 ms accumulation time per spectrum. A maximum of 20 precursors per cycle were chosen for fragmentation from each MS spectrum with100 ms minimum accumulation time for each precursor and dynamic exclusion for 20 s. Tandem mass spectra were recorded in high sensitivity mode (resolution > 15,000) with rolling collision energy on and iTRAQ reagent collision energy adjustment on.

### Tissue collection and morphological analysis

The ovaries were fixed in 4% formaldehyde for 24 h, embedded in paraffin wax, sectioned at 4 μm thickness, and mounted on glass slides for hematoxylin and eosin (H&E) staining. At least five sections (taken 100 μM apart) from an ovary and 5 ovaries from each group were photographed for follicle assessment. A follicle was deemed present if the oocyte contained a germinal vesicle, and was counted and classified according to its health and developmental stage, as a healthy (including primordial, primary, secondary, and antral follicle) or atresia follicle, according to previously described criteria [20].

### Immunofluorescence

For the immunofluorescence analysis, the sections were incubated with the antibodies summarized in Additional file 1: Table S1, followed by incubation with Alexa-Fluor-488-labeled secondary antibodies (Molecular Probes, MA, USA) and 4′,6-diamidino-2-phenylindole (Invitrogen, Carlsbad, CA, USA) to visualize the nuclei. The immunofluorescent images were obtained using FluoView FV1000 confocal microscopy (Olympus, Tokyo, Japan).

### TUNEL analysis

Cell apoptosis in the follicles was evaluated in sections using a terminal-deoxynucleotidyltransferase-mediated dUTP nick end labeling (TUNEL) assay for the situ visualization of DNA fragmentation with the commercial DeadEnd™ Fluorometric TUNEL System (Promega, Madison, WI, USA). Images were obtained with a FluoView FV1000 confocal microscope. Every 25th section in 5 ovaries from each group were analyzed, and the level of apoptosis was expressed as the total number of apoptotic cells on five sections from an ovary of each mouse.

### Serum hormone measurement with enzyme-linked immunosorbent assays (ELISAs)

The mice were anesthetized to collect their blood via cardiocentesis. The blood samples were centrifuged at 3000×g for 10 min and the serum collected. The plasma levels of FSH and estradiol (E2) hormone were measured with ELISA kits (Elabscience Biotechnology, Wuhan, China).

### Real-time quantitative PCR

Total ovarian RNA was purified with TriGene Reagent, and then processed to cDNA with the RETROscript Reverse Transcription Kit, and amplified and quantified with the RealStar Power SYBR Kit (all from GenStar BioSolutions, Beijing, China) with a StepOnePlus Real-Time PCR System (Applied Biosystems, Waltham, MA, USA). The gene-specific primers (Sangong Biotech, Shanghai, China) for qPCR were: For *Hspa5*, forward: 5′-ACTTGGGGACCACCTATTCCT-3′, reverse: 5′-GTTGCCCTGATCGTTGGCTA-3′; for *Hsp90ab1*, forward: 5′- GTCCGCCGTGTGTTCATCAT-3′, reverse: 5′-GCACTTCTTGACGATGTTCTTGC-3′. The expression of *Hspa5* and *Hsp90ab1* were normalized to that of glyceraldehyde 3-phosphate dehydrogenase (*Gapdh*).

### Western blotting

Ovaries were homogenised in lysis buffer and boiled in sodium dodecyl sulfate (SDS) loading buffer. The protein extracts were then subjected to 6–12% SDS-PAGE and electrotransferred to nitrocellulose membranes (GE Healthcare Life Sciences, Beijing, China). The membranes were blocked with 5% nonfat dry milk for 1 h at room temperature, washed, and incubated with the indicated primary antibody at 4 °C overnight. The membranes were further washed, incubated with Peroxidase-AffiniPure Goat Anti-Rabbit or -Mouse IgG (H + L) (Jackson ImmunoResearch, West Grove, PA, USA) for 1 h at room temperature, washed again, and finally visualized with an enhanced chemiluminescence kit (PerkinElmer, Waltham, MA, USA). The primary antibodies used for the immunoblotting analysis are summarized in Additional file 1: Table S1.

### In vivo inhibitor treatment

Seventy-two mice were evenly and randomly divided into four groups, which received intraperitoneal injections of 0.9% saline as control group, and experimental groups were intraperitoneal injections of CDDP (2.5 mg/kg/d), CDDP + 4-PBA (2.5 + 150 mg/kg/d, respectively) [21], and CDDP + 3-methyladenine (3-MA; 2.5 + 10 mg/kg/d), respectively [22] (4-PBA and 3-MA were from Sigma-Aldrich), for 1, 3 and 7 days. The mice were euthanized at the indicated time points, and their ovaries were isolated for histological analysis and a western blotting assay.

### Cell culture and related assays

#### Cell culture

KGN cell line (a generous gift from Prof. Yiming Mu, Chinese PLA General Hospital, China), which was established from a human ovarian granulosa cell tumor [23], and COV434 cell line (a generous gift from Prof. Hongyan Yang, Second Affiliated Hospital of Guangzhou University of Traditional Chinese Medicine, China), immortalized granulosa cells derived from a solid granulosa cell tumor [24], were cultured with Dulbecco’s Modified Eagle’s Medium/Ham’s F12 (Invitrogen) supplemented with 10% fetal bovine serum (FBS, Gibco by Invitrogen). The cells were maintained in a subconfluent state at 37 °C during all the experiments in an atmosphere of 5% CO2/95% humidified air.

#### siRNA transfection

KGN and COV434 cells (1 × 10^5^) were seeded in 12-well plates for 24 h, and then transfected with siRNA (GenePharma, Shanghai, China) targeting *HSPA5* and *HAP90AB1* mRNAs using Lipofectamine 3000 (Invitrogen). After 48 h, the cells were exposed to CDDP (50 μM) for the indicated times for western blotting. The siRNA sequences were: *HSPA5*: sense: 5′-UGUUGGUGGCUCGACUCGATT-3′, antisense: 5′-UCGAGUCGAGCCACCAACAAG-3′; *HSP90AB1*: 5′-AGUAAACUAAGGGUGUCAATT-3′, antisense: 5′-UUGACACCCUUAGUUUACUGC-3′. Nonspecific siRNA sequences were used as the negative controls (NC): sense: 5′-UUCUCCGAACGUGUCACGUTT-3′, antisense: 5′-ACGUGACACGUUCGGAGAATT-3′.

#### Cell apoptosis analysis with flow cytometry

Apoptotic cells were measured with an Annexin V/ propidium iodide (PI) apoptosis analysis kit (Sungene Biotech, Tianjin, China). Briefly, 1 × 10^6^ cells were seeded in six-well plates and then incubated with the indicated chemicals for the indicated times. After the cells were washed with cold phosphate-buffered saline, they were collected by centrifugation and the cell concentration was adjusted to 1 × 10^6/^mL with binding buffer. Annexin V solution (5 μL) was added to the tubes and incubated for 10 min, followed by incubation with 5 μL of PI solution for another 5 min. The whole operation was performed at room temperature and protected from light. The final fluorescence was measured with a flow cytometer (Beckman Coulter, Brea, CA, USA), and a CytExpert software was used for data acquisition (1 × 10^4^ events per sample). The percentages of cells in four classes were determined: viable (annexin V^neg^, PI^neg^), early apoptosis (annexin V^pos^, PI^neg^), late apoptosis (annexin V^pos^, PI^pos^), and necrosis (annexin V^neg^, PI^pos^).

#### Autophagy detection with DALGreen staining

Cells were plated in confocal dishes and cultured at 37 °C with 1 mL of a 0.6 μM DALGreen [25] (Dojindo Laboratories, Shanghai, China) working solution for 30 min. After the cells were washed twice with culture medium, they were incubated with 50 μM CDDP, with or without 5 mM 4-PBA [26] or 5 mM 3-MA [27]. 8 h later, cells were washed twice with Hanks’ HEPES buffer and observed with a FluoView FV1000 confocal microscope. Cells with more than three DALGreen-positive foci were considered to be autophagy-positive cells.

### Statistical analysis

The peptide data were analyzed using Protein Pilot Software 4.0 (AB SCIEX, CA), and the parameters were set as follows: Cys alkylation: MMTS; ID focus: biological modifications; Digestion: typsin; Database: Uniprot_MOUSE; Search effort: thorough ID. All experiments were performed at least in duplicate. Data are presented as means ± SD, and differences between groups were analyzed with an independent-samples *t*-test and among groups with one-way ANOVA followed by the SNK test (SPSS 25.0, SPSS Inc., Chicago, IL, USA). If the data were not normally distributed, the nonparametric Mann-Whitney test was used. A χ2 test was used for rate comparisons. *p* < 0.05 was considered statistically significant.

## Results

### Ovarian HSPA5 and HSP90AB1 protein levels were significantly reduced in the CDDP-induced POI model

**Fig. 1:**
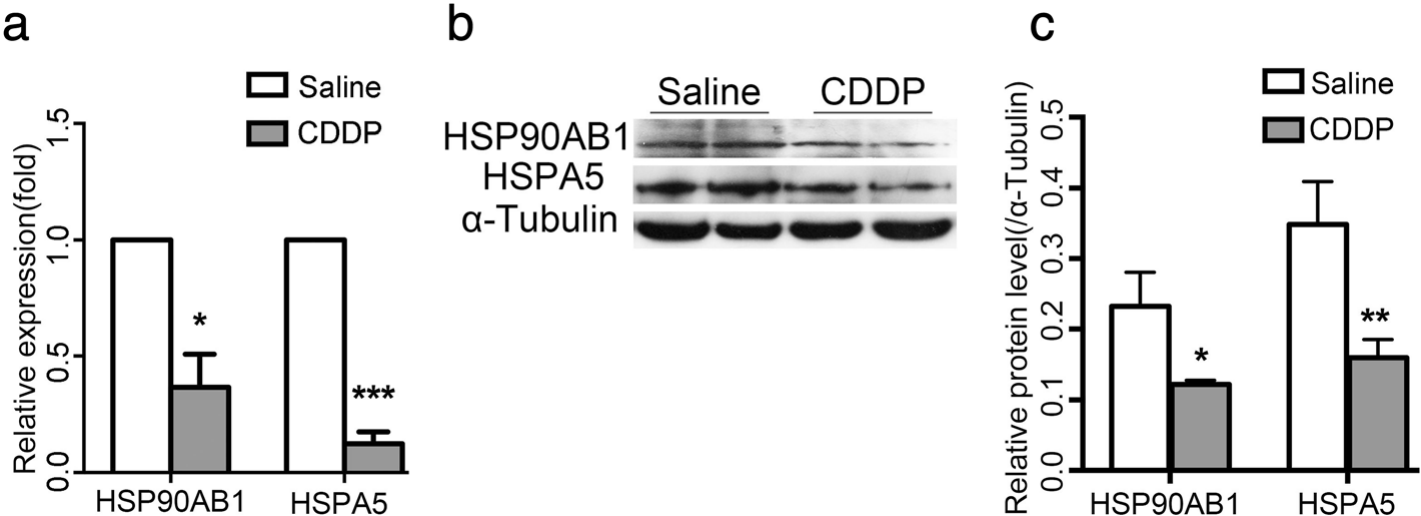
CDDP decreases the expressions of HSPA5 and HSP90AB1 in ovaries. **a** qPCR showed that both mRNA levels of *Hspa5* and *Hsp90ab1* were decreased in CDDP-treated ovaries. **b** western blotting showed that both protein levels of HSPA5 and HSP90AB1 were decreased in CDDP-treated ovaries. **c** Quantification of the results in b. Protein levels were normalized to that of Tubulin. Data are presented as mean ± SD. *n* = 3, * *P* < 0.05, ** *P* < 0.01, *** *P* < 0.001

To investigate the molecular changes underlying CDDP-induced ovarian damage, we generated a mouse model of POI by injecting mice intraperitoneally with CDDP for 7 days, and then subjecting their ovaries to proteomic screening with an iTRAQ analysis. The expression levels of 214 proteins were significantly upregulated (fold change ≥1.5) and the levels of 180 proteins were significantly downregulated (fold change ≥ − 1.5) (Additional file 1: Table S2). Of these 394 differentially expressed proteins, HSPA5 and HSP90AB1, two well-established markers for ERS and UPR [14], were of our particular interest, because they were downregulated 2.5-fold and 2.3-fold, respectively, in the CDDP-treated ovaries (Table 1). Furthermore, a STRING analysis showed that both of them were at the core of signaling network (Additional file 1:Figure S1). To confirm this proteomic result, we used qPCR to show that the relative mRNA expression of *Hspa5* and *Hsp90ab1* was significantly lower in the CDDP group than in the control group (Fig. 1a). Western blotting further confirmed the reduction in their protein levels in the CDDP group (Fig. 1b, c). Although the proteomic analysis indicated a more dramatic decline in the protein levels of FADS2 and HSD11B2, which, however, were not confirmed by western blotting (Additional file 1:Figure S2). These results indicated that ERS was strongly associated with CDDP-induced ovarian damage.

**Table 1:** iTRAQ analysis showed reduced HSPA5 and HSP90AB1 expression in a CDDP-induced POI model

Name	Gene Name	Fold Change^a^
78 kDa glucose-regulated protein	GN=HSPA5	−2.5
Heat shock protein HSP 90-beta	GN=HSP90AB1	−2.3

^a^“-” = Downregulated in the CDDP-treated ovaries. *n* = 3

### Inhibition of ERS attenuates CDDP-induced granulosa cells autophagy and apoptosis

**Fig. 2:**
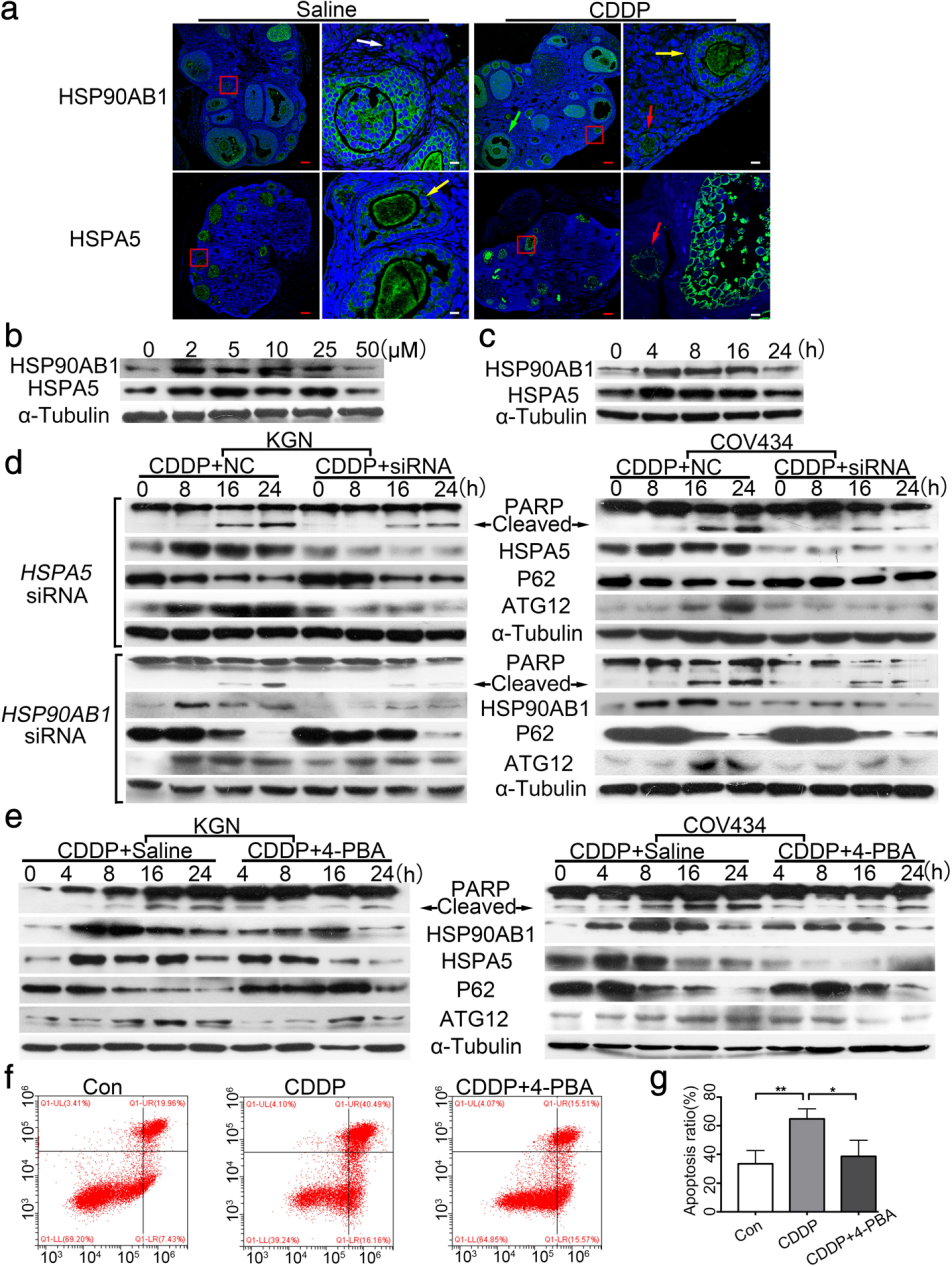
Inhibition of ERS depresses CDDP-induced ERS, autophagy and apoptosis in granulosa cells. **a** Immunofluorescence of HSP90AB1 and HSPA5 in the saline and CDDP-treated ovaries. Nuclei were stained with DAPI. White arrow indicates primordial follicle; red, primary follicle; yellow, secondary follicle; green, antral follicle. Red scale bars = 80 μm, White scale bars = 10 μm (**b**) Immunoblotting of HSP90AB1 and HSPA5 in the KGN cells treated with CDDP at indicated concentrations (0, 2, 5, 10, 25 and 50 μM) for 24 h. **c** Immunoblotting of HSP90AB1 and HSPA5 in the KGN cells treated with CDDP at 50 μM for indicated times (0, 4, 8, 16, 24 h). **d** KGN and COV434 cells were transfected with *HSPA5* and *HSP90AB1*-specific siRNA and NC-siRNA respectively for 48 h, and then treated with 50 μM CDDP for indicated times. Immunoblotting was carried out to detect the protein levels of ERS-, autophagy- and apoptosis-related genes. **e** KGN and COV434 cells were treated with 50 μM CDDP with/without 5 mM 4-PBA for indicated times. ERS, autophagy and apoptosis levels were detected by western blot. **f** KGN cells were treated with 50 μM CDDP with/without 5 mM 4-PBA for 24 h and then stained with Annexin V-FITC and PI. Both cells at early (annexin V^pos^; PI^neg^) and late apoptotic stages (annexin V^pos^; PI^pos^) were counted. **g** Quantification of the results in f. * *P* < 0.05, ** *P* < 0.01

We next characterized the expression profiles of HSPA5 and HSP90AB1 in the CDDP-treated ovaries with immunofluorescence. Both HSPA5 and HSP90AB1 were predominantly localized to the cytoplasm around the nucleic of granulosa cells from secondary and antral follicles, modestly expressed in those from primary follicles, while hardly present in those of from primordial follicles (Fig. 2a), indicating that ERS in granulosa cells may play a role in CDDP-induced follicle loss. Therefore, two human granulosa tumor cell lines, KGN and COV434, were used for an in vitro study. CDDP at 50 μM significantly decreased the proteins expression of HSPA5 and HSP90AB1 in KGN cells at 24 h,thus we treated cells with the dose in the following cell experiments (Fig. 2b). CDDP (50 μM) induced time-dependent changes in HSPA5 and HSP90AB1 protein levels in the KGN cells, with an increase at 4 h, which were then decreased at 24 h (Fig. 2c), indicating that ERS was induced upon exposure to CDDP. Autophagy related 12 (ATG12) is positively involved in the assembly of the autophagosome. P62 binds to polyubiquitinated proteins and LC3 on the autophagosomal membrane to target aggregates to the autophagosomes for degradation, and its expression negatively correlates with autophagosome formation [28]. CDDP treatment induced a time-dependent increase in the expression of ATG12 and a reduction in P62, and also an increase in cleaved PARP protein, a marker of cell apoptosis (Fig. 2d). Interestingly, when either the *HSPA5* or *HSP90AB1* gene was knocked down with RNA interference (RNAi), the levels of both ATG12 and cleaved PARP proteins were greatly reduced (Fig. 2d). This finding suggested that ERS induced cell autophagy and apoptosis in response to excessive CDDP treatment. This was verified by introducing 4-PBA, an ERS inhibitor, into CDDP-treated cells, where it significantly relieved CDDP-induced ERS, cell autophagy, and cell apoptosis (Fig. 2e). A flow-cytometric analysis of annexin V/PI staining further confirmed that CDDP-induced cell apoptosis was largely prevented by 4-PBA (*P* < 0.05, Fig. 2f, g). These results together demonstrated that the alleviation of ERS attenuated CDDP-induced cell autophagy and apoptosis.

### Suppressing autophagy with 3-MA neither reduced CDDP-induced cell apoptosis nor alleviated ERS

**Fig. 3:**
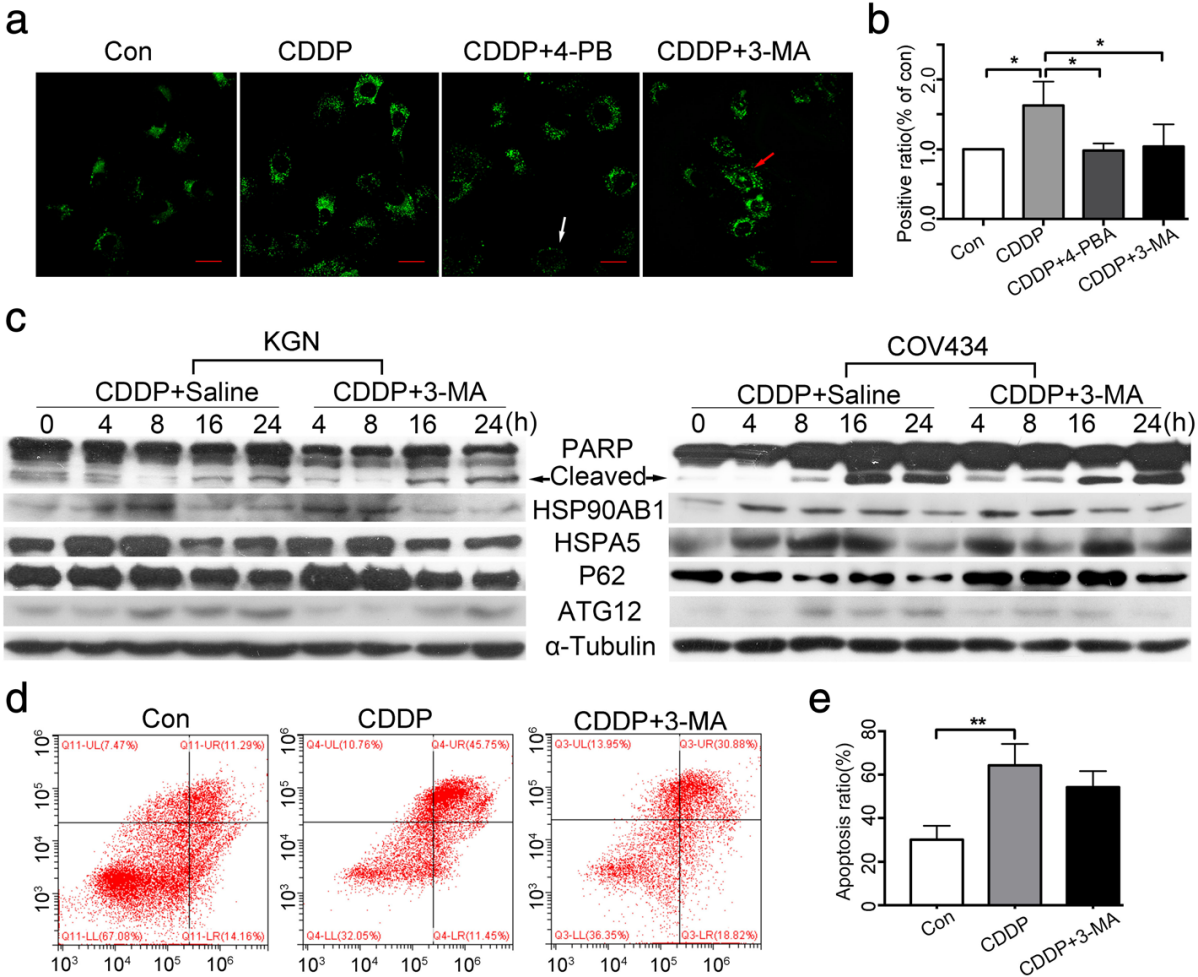
Suppressing autophagy hardly alleviates CDDP-induced cell apoptosis or ERS. **a** Autophagy levels in KGN cells after indicated treatments by DALGreen staining. Positive autophagosomes were observed by fluorescence microscopy. White arrow indicates autophagosome-negative cell and the red indicates autophagsome-positive cell. Scale bars = 80 μm. **b** Quantification of the positive phagosomes in a. **c** KGN and COV434 cells were treated with 50 μM CDDP with/without 5 mM 3-MA for indicated times. ERS, autophagy and apoptosis levels were detected by western blotting. **d** KGN cells were treated with 50 μM CDDP with/without 5 mM 3-MA for 24 h and then stained with Annexin V-FITC and PI. **e** Quantification of the results in d. * *P* < 0.05

We next examined whether inhibiting autophagy protected cells from CDDP-induced apoptosis. DALgreen staining showed that although both 3-MA and 4-PBA efficiently reduced the production of CDDP-induced autophagosomes (*P* < 0.05) (Fig. 3a, b). Western blotting also showed that 3-MA alleviated CDDP-induced autophagy level by reducing the expression of ATG12 and by increasing that of P62, whereas the protein levels of cleaved PARP, HSPA5, and HSP90AB1 remained stable (Fig. 3c). The finding that 3-MA negligibly reduced CDDP-induced cell apoptosis was further supported by the results of flow cytometry (*p* > 0.05, Fig. 3d, e). These findings collectively suggested that lowering the autophagy level with 3-MA neither reduced cell apoptosis nor alleviated ERS during CDDP treatment.

### Effects of 4-PBA and 3-MA on CDDP-induced POI in vivo

**Fig. 4:**
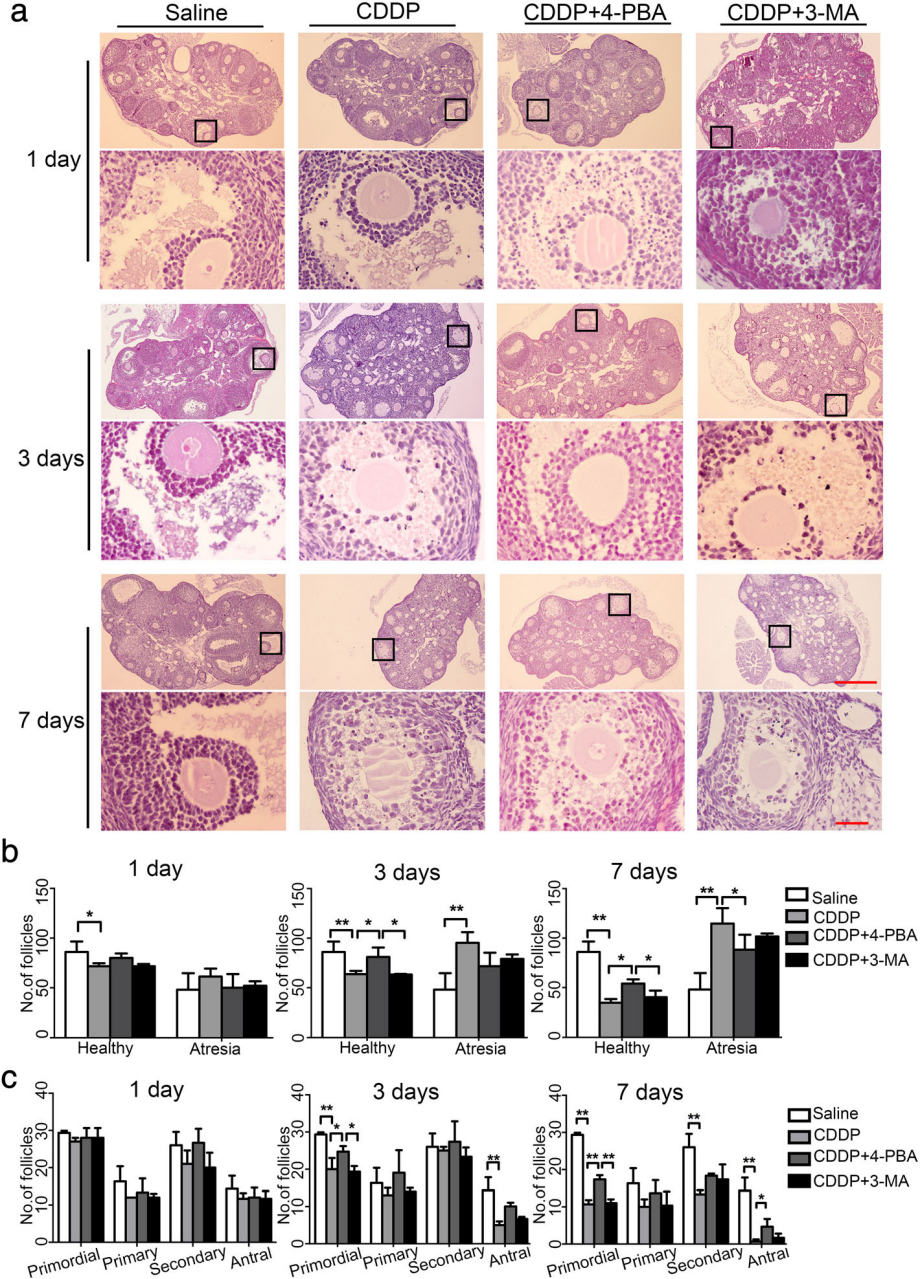
Effects of 4-PBA and 3-MA on ovarian histology in CDDP-treated mice. Mice were treated with saline, CDDP, and CDDP with 4-PBA or 3-MA for 1 day, 3 days and 7 days, respectively. **a** H&E staining showed the ovarian histology in each group. Five sections (taken 100 μm apart) from an ovary were photographed for follicle assessment. Scale bars = 40 μm (upper panels) and 4 μm (lower panels). **b** Comparison of the total number of healthy and atresia follicles among groups. Healthy follicles include healthy primordial, primary, secondary and antral follicles. **c** Comparison of the number of healthy primordial, primary, secondary and antral follicles among groups. *n* = 5, * *P* < 0.05, ** *P* < 0.01

**Fig. 5:**
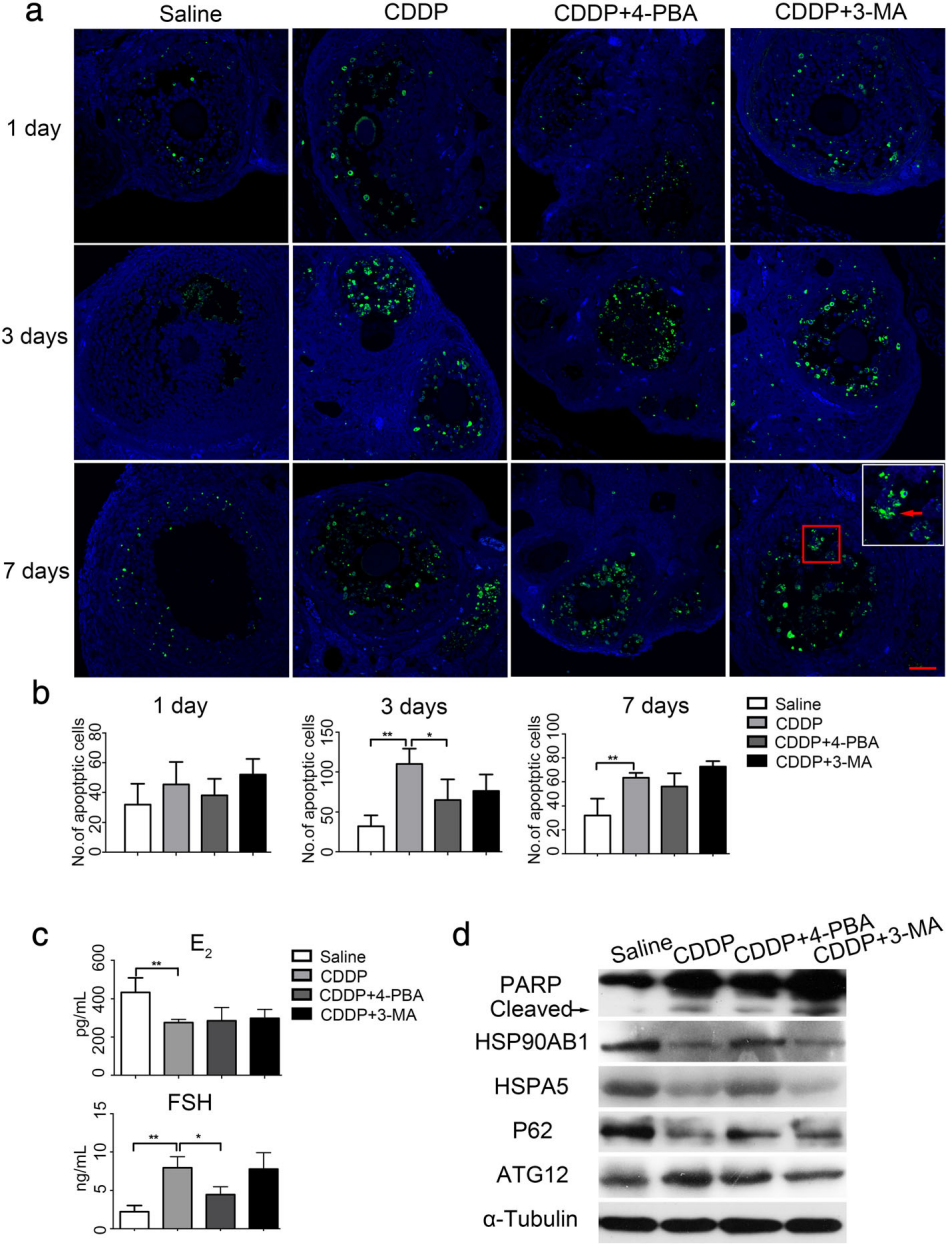
Effects of 4-PBA and 3-MA on ovarian function in CDDP-treated mice. **a** Granulosa cell apoptosis in ovarian sections from each group was measured by fluorescent TUNEL staining. Green fluorescences indicate TUNEL-positive apoptotic cells (red arrow). The level of apoptosis is present as the total number of apoptotic granulosa cell on five sections (taken 100 μm apart) from an ovary. Scale bars =40 μm. **b** Quantification of TUNEL-positive apoptotic cells in each group. **c** Plasma E_2_ and FSH levels by ELISA in mice with indicated treatment for 7 days. **d** Immunoblotting of the protein levels of ERS-, autophagy- and apoptosis-related proteins in each group. Protein extractions were from mice with indicated treatment for 7 days. *n* = 5, * *P* < 0.05, ** *P* < 0.01

We then compared the protective effects of 4-PBA and 3-MA against CDDP-induced ovarian damage in vivo. Female mice were treated with CDDP combined with 4-PBA or 3-MA for 1, 3, or 7 days. A histological examination showed that the morphologies of follicles at different stages of development were parallel across all the groups on day 1, except that the healthy follicles in CDDP group decreased, compared with the saline group ((*P* < 0.05, Fig. 4a-c). By day 3, the CDDP-treated group had significantly fewer healthy follicles and more atresia follicles than the saline-treated group (*P* < 0.01), which was predominantly attributable to the loss of primordial and antral follicles (Fig. 4b, c). By day 7, the longer exposure to CDDP seriously damaged the healthy follicles and produced excessive atresia follicles than the saline-treated group (both *P* < 0.01). Besides primordial and antral follicles, more secondary follicles were damaged, whereas the primary follicles appeared not susceptible. We speculate that it is associated with follicular recruitment and CDDP-induced granulosa cell apoptosis in growing follicles. The more granulosa cells in the growing population of follicles, the more susceptible they were to CDDP. These data support a previous report that chemotherapeutic agents over activate the primordial follicles and damage the growing population of follicles, leading to the premature depletion of follicle reserves [29]. As expected, 4-PBA, but not 3-MA, markedly ameliorated CDDP-induced follicle loss and preserved more healthy follicles by protecting follicles from atresia (Fig. 4b, c). Consistent with this, a TUNEL assay showed that CDDP-induced granulosa cell apoptosis peaked on day 3 (*P* < 0.01), and was markedly ameliorated by 4-PBA (*P* < 0.05) but not by 3-MA (*P* > 0.05, Fig. 5a, b). By day 7, the intensity of granulosa cell apoptosis had decreased in the CDDP group compared to the 3rd day, and 4-PBA slightly reduced the number of apoptotic cells (Fig. 5a, b). Consistent with the histological results, hormone measurements with ELISAs showed that exposure to CDDP for 7 days clearly reduced the plasma E_2_ content, and especially, increased the FSH level (both *P* < 0.01, Fig. 5c), which is considered as the most important biochemical indicator of POI in clinical. The 4-PBA treatment clearly-inhibited the CDDP-induced increase in FSH, but not the reduction in E_2_, whereas and the 3-MA treatment had negligible effects on these changes (Fig. 5c). We propose that the secretion of FSH is affected by the negative feedback of E_2_ and FSH receptors, the production of E_2_ decreased due to excessive granulosa cell apoptosis induced by CDDP, which in turn stimulates the secretion of FSH from pituitary. 4-PBA reduced granulosa cell apoptosis and may preserve FSH receptors, therefore, the secretion of FSH was not increased significantly. Furthermore, an immunoblotting analysis of whole-mount ovarian proteins showed that in vivo treatment with 4-PBA blocked the increases in the protein levels of ATG12 and cleaved PARP, but not of HSPA5 and HSP90AB1during CDDP treatment (Fig. 5d). This may indicate different responses to 4-PBA between in vivo and in vitro. The CDDP-induced increase in cleaved PARP was resistant to 3-MA treatment, although 3-MA reduced the protein level of ATG12 and increased P62 (Fig. 5d). These results together provided in vivo evidence supporting the notion that the alleviation of ERS attenuated CDDP-induced granulosa cell death and ovarian damage.

## Discussion

**Fig. 6:**
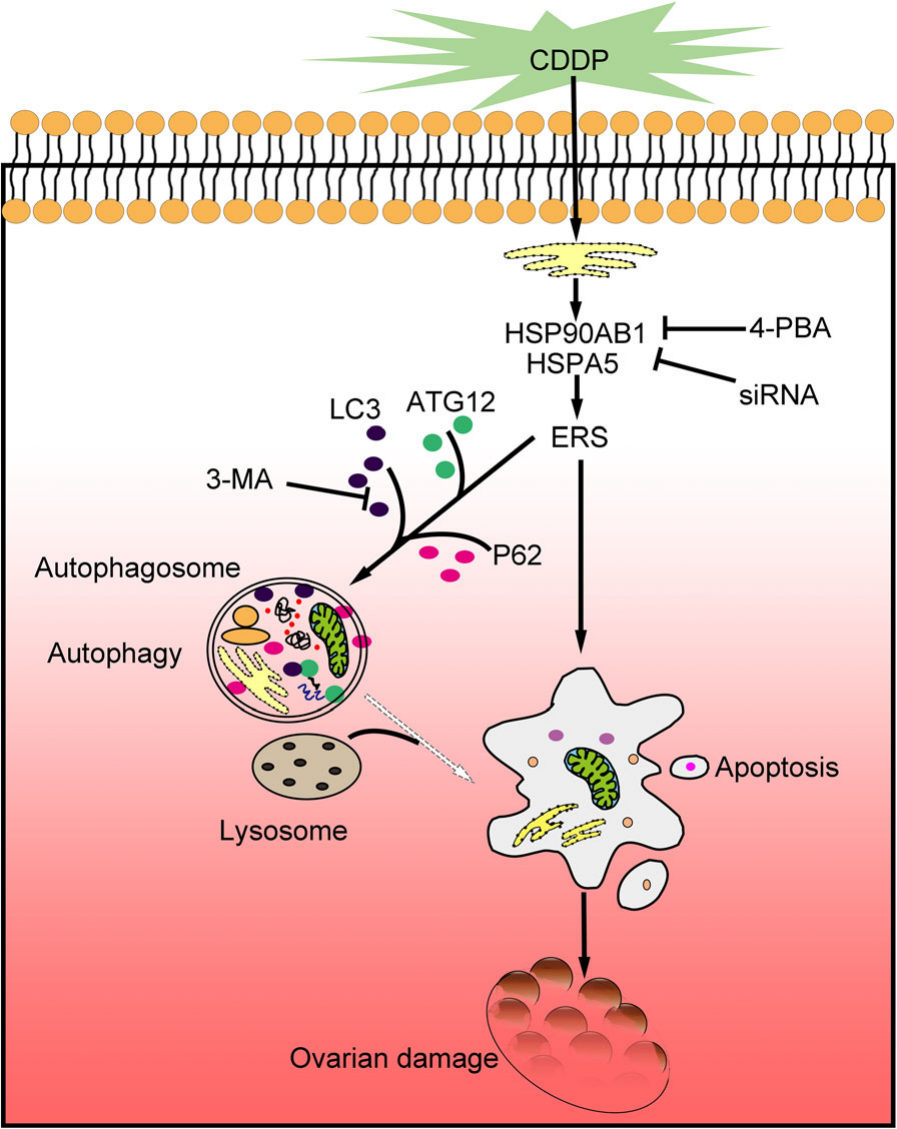
A schematic mechanism of ERS in CDDP-induced ovarian damage. Exogenous CDDP increases the accumulation misfolded proteins in ER in granulosa cells, which subsequently enhance HSPA5 and HSP90AB1 expressions, leading to activation of ERS. ERS promotes cell autophagy and apoptosis. Excessive granulosa cell apoptosis induces follicular atresia contributing to ovarian dysfunction. 4-PBA can alleviate ERS, suppress cell autophagy and apoptosis, preserve follicles, and thus prevent against CDDP-induced ovarian damage

In this study, we established the central role of ERS in CDDP-induced granulosa cell apoptosis. Reducing ERS by knocking down the expression of either the *HSPA5* or *HSP90AB1* gene with RNAi or by introducing the ERS inhibitor 4-PBA significantly attenuated CDDP-induced cell autophagy and apoptosis in cultured KGN and COV434 cells. However, inhibiting cell autophagy with 3-MA negligibly blocked the changes in ERS and apoptosis induced by CDDP (Fig. 6). Our in vivo experiments also demonstrated that in vivo treatment with 4-PBA, but not 3-MA, prevented CDDP-induced follicle loss and hormone dysregulation. Therefore, the alleviation of ERS protects against cisplatin-induced granulosa cell apoptosis and ovarian damage.

Our results clearly demonstrated that ERS was an important switch in the decision of the granulosa cell fate during CDDP treatment. An iTRAQ analysis revealed a notable decline in both the HSPA5 and HSP90AB1 proteins, which was confirmed with real time quantitative PCR and immunoblotting. However, in vitro experiments using either KGN or COV434 cells showed that the levels of both the HSPA5 and HSP90AB1 proteins increased at 4 h, and declined at 24 h after treatment with CDDP. These changes in response to CDDP treatment prompted us to infer that CDDP induced misfolded or unfolded proteins accumulated on the ER, and activated the UPR to upregulate ER chaperones such as HSPA5 and HSP90AB1, which degraded the misfolded or unfolded proteins [30]. Autophagy was subsequently prompted to degrade the denature proteins and damaged organelles in autolysosomes, manifested as an increase in ATG12 and a decrease in P62 at 8 h after treatment with CDDP. This may be a protective mechanism by which ERS maintains cellular homeostasis, possibly by triggering prosurvival cellular events. However, when stress persists, ERS elements are impaired and cellular homeostasis is perturbed, ultimately leading to apoptosis [31], as indicated in the results that cleaved PARP increased and ERS-related proteins HSPA5 and HSP90AB1 decreased. 4-PBA reduced granulosa cell apoptosis by alleviating the prolonged or excessive ERS in granulosa cell, depressing the expression of HSPA5 and HSP90AB1, attenuating the autophagy level. It is interesting to note that although the expression of HSPA5 and HSP90AB1 is weak in primordial follicles, PBA treatment also effectively preserves primordial follicles, meaning that CDDP may acts in primordial and developed follicles via different mechanisms, and ERS may not be the only target for PBA.

We also investigated whether cell autophagy was one of the mechanisms by which ERS maintained cellular homeostasis. Autophagy degrades destructive substances and dysfunctional organelles to maintain cellular homeostasis. In cultured KGN and COV434 cells, exposure to CDDP induced ERS as early as 4 h, which was followed by autophagy at about 8–16 h, and finally excessive apoptosis at about 24 h. Therefore, we hypothesized that CDDP-induced ERS stimulated autophagy, which in turn degraded damaged ER elements to maintain the survival of the cell. However, excessive or prolonged ERS and autophagy will ultimately lead to apoptosis. This hypothesis is supported by the finding that the suppression of ERS, either with siRNA targeting *HSPA5* or *HSP90AB1* or with 4-PBA, significantly inhibited cell autophagy and apoptosis, which eventually alleviated follicle loss. However, although 3-MA reduced the level of autophagy, it negligibly attenuated ERS or apoptosis. A previous study indicated that ERS inhibits autophagy [18] and another study showed that CDDP combined with an autophagy inhibitor or an ERS inhibitor increased the apoptosis of human lung cancer cells [32]. We suspect that these discrepancies may be attributable to the different doses of CDDP used, the different cell types examined, and therefore the different sensitivities of the cells to CDDP.

Various rescue methods have been tested to restore chemotherapy-induced ovarian damage, including human amniotic epithelial cells [33], co-treatment with a gonadotropin-releasing hormone agonist [34], melatonin treatment [19], and umbilical cord mesenchymal stem cells [35, 36]. However, the effectiveness has not been confirmed in the ovarian function and fertility when these treatments have been applied in clinical practice. As far as we know, this is the first report to demonstrate a relationship between ERS, autophagy, and apoptosis both in vitro and *vivo*. In vitro, 4-PBA alleviated ERS and attenuated ERS-induced autophagy and apoptosis in granulosa tumor cells. In a mouse model, 4-PBA played a protective role against CDDP-induced ovarian dysfunction. Based on these results, we considered that ERS plays a vital role in regulating the growth of granulosa cell and the development of the ovary. Therefore, 4-PBA could be an effective agent for relieving CDDP-induced ovarian damage, while the present evidences do not support that relieving autophagy can attenuate ERS or apoptosis. A previous study showed that 3-MA led to the simultaneous attenuation of autophagy and apoptosis, but did not affect cell necrosis [37]. This discrepancy reminded us that rescuing ovarian injury by inhibiting autophagy should be undertaken with caution.

## Conclusions

In summary, we have clarified the relationships between ERS, autophagy, and apoptosis in CDDP-induced granulosa cell apoptosis and ovarian damage, both in vitro and in vivo. Alleviating ERS attenuated CDDP-induced autophagy and apoptosis. The 4-PBA largely attenuates CDDP-induced granulosa cell apoptosis and ovarian damage. Overall, our results can be used as a reference point in determining the underlying pathophysiology of ovarian injury induced by chemotherapeutic treatments and may provide potential pharmacotherapeutic options.

## Additional file

Additional file 1 **Figure S1.** A STRING analysis shows signaling network. **Figure S2.** The protein levels of FADS2 and HSD11B2 by western blotting. **Figure S3**. Full-length western blot images with negative controls. **Table S1.** Antibodies used for different experiments in this study. **Table S2.** down-regulated 1.5-fold proteins (PDF 7703 kb)

## Abbreviations

3-MA: 3-methyladenine
4-PBA: 4-phenylbutyric acid
CDDP: Cisplatin
E2: Estradiol
ELISAs: Enzyme-linked immunosorbent assays
ERS: Endoplasmic reticulum stress
FBS: Fetal bovine serum
FSH: Follicle-stimulating hormone
H&E: Hematoxylin and eosin
HSP90AB1: Heat shock protein HSP 90-beta
HSPA5: 78-kDa glucose-regulated protein
iTRAQ: Isobaric tags for relative and absolute quantification
PI: Propidium iodide
POF: Premature ovarian failure
POI: Premature ovarian insufficiency
qPCR: Real-time quantitative PCR
RNAi: RNA interference
TUNEL: Terminal-deoxynucleotidyltransferase-mediated dUTP nick end labeling
UPR: The Unfolded protein response
