# Supplementary material for: Alleviation of endoplasmic reticulum stress protects against cisplatin-induced ovarian damage
Source: Reprod Biol Endocrinol. 2018 Sep 3;16:85. doi: 10.1186/s12958-018-0404-4 (PMC6122480; doi:10.1186/s12958-018-0404-4)
Supplement: Supplementary file 1 — Figure S1. A STRING analysis shows signaling network. Figure S2. The protein levels of FADS2 and HSD11B2 by western blotting. Figure S3. Full-length western blot images with negative controls. Table S1. Antibodies used for different experiments in this study. Table S2. down-regulated 1.5-fold proteins (PDF 7703 kb) [file 12958_2018_404_MOESM1_ESM.pdf]

Additional Figure 1

A STRING analysis shows signaling network.

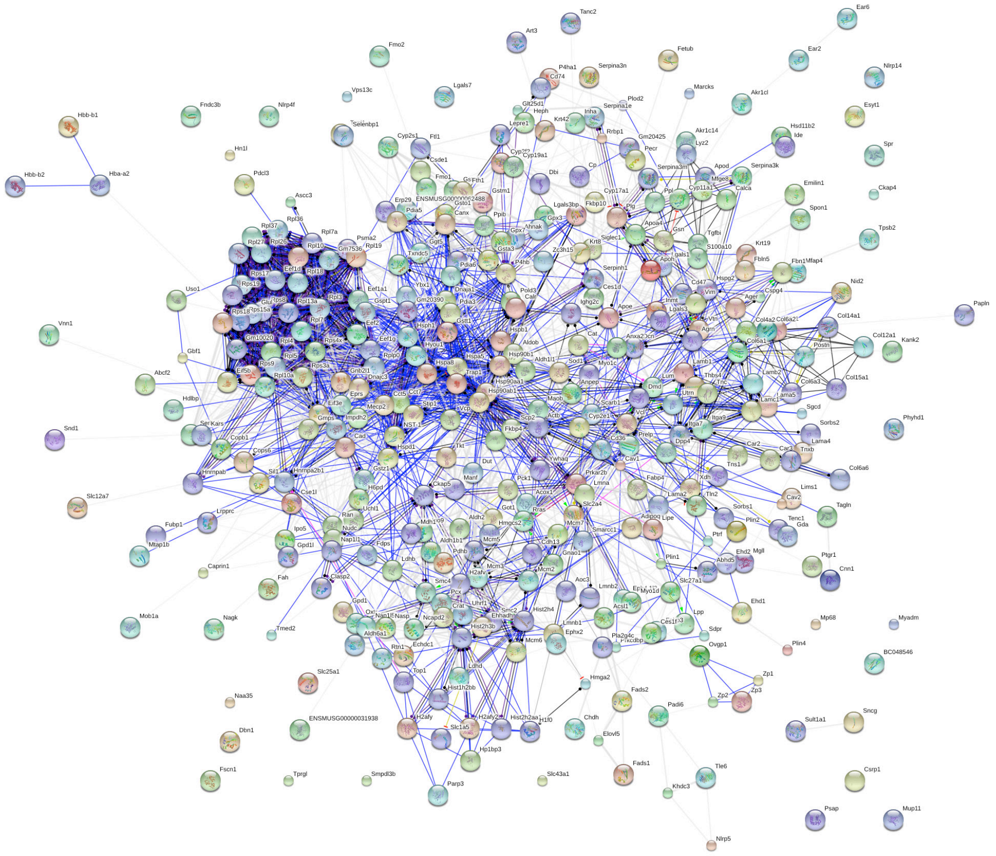

Additional Figure 2

The protein levels of FADS2 and HSD11B2 by western blotting.

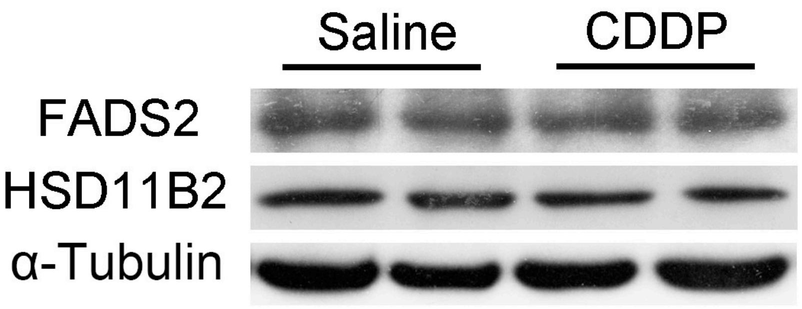

Additional Figure 3

Full-length western blot images with negative controls

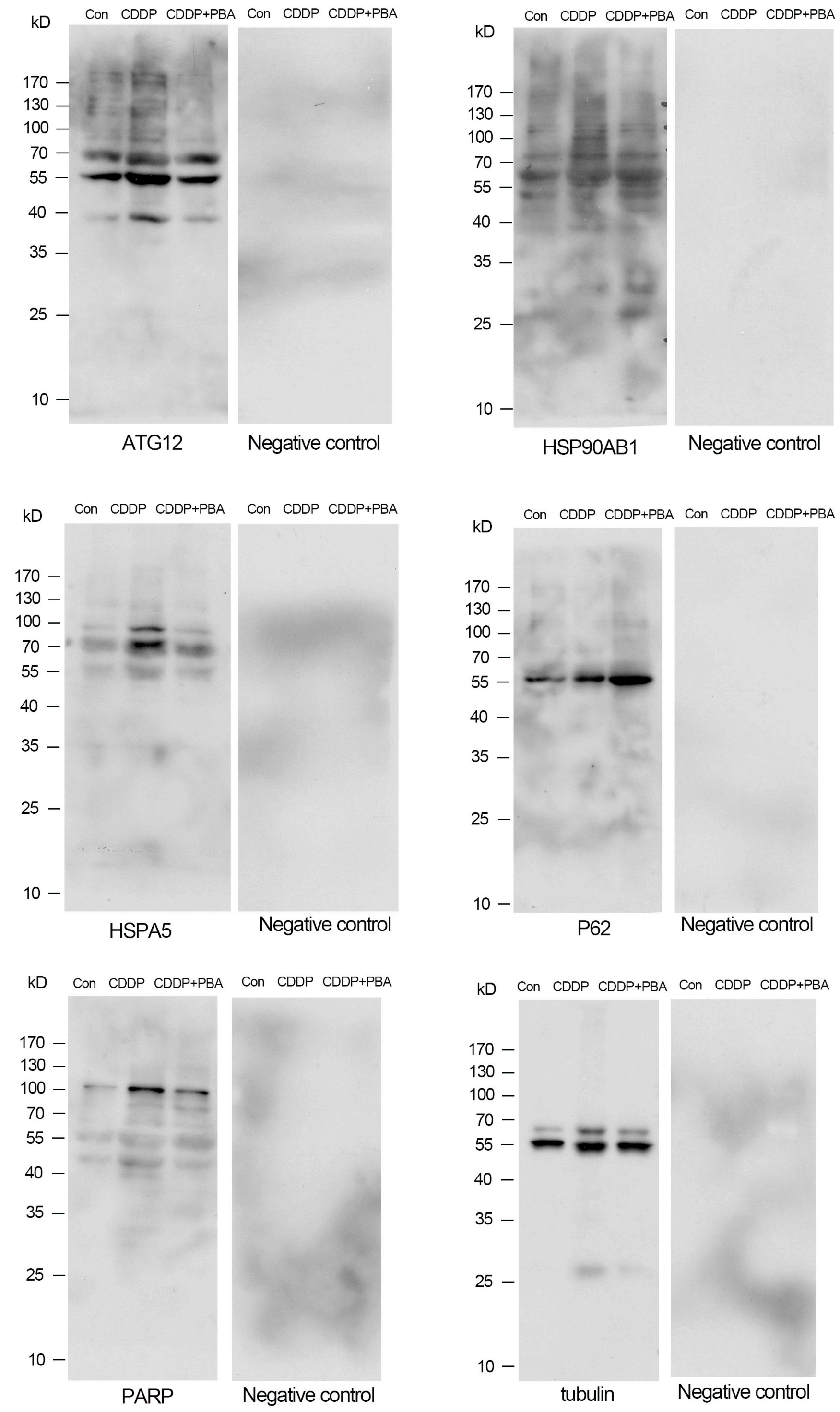

Additional file : Table 1

Antibodies used for different experiments in this study.

Antibody	Vendor	Catalog number	Application	Working dilution for WB or (IF)
Rabbit anti-HSPA5	ABclonal	A11366	WB, IF	1:1000, (1:100)
Rabbit anti-HSP90AB1	Zen Bioscience	616474	WB, IF	1:1000, (1:100)
Rabbit anti-P62	Zen Bioscience	380612	WB	1:1000
Rabbit anti-PARP	Cell Signaling Technology	#9532	WB	1:1000
Rabbit anti-ATG12	Biowold	AP0758	WB	1:2000
Mouse anti-α-Tubulin	Sungenen	KM9007	WB	1:5000

WB, western blot; IF, immunofluorescence.

Additional file : Table 2

down-regulated 1.5-fold proteins

N	Citable Accession	Name	Gene Name	Fold Change
1	Q9Z0R9	Fatty acid desaturase 2 OS=Mus musculus GN=Fads2 PE=1 SV=1	GN=Fads2	10.15
2	Q61009	Scavenger receptor class B member 1 OS=Mus musculus GN=Scarb1 PE=1 SV=1	GN=Scarb1	8.05
3	Q62151	Advanced glycosylation end product-specific receptor OS=Mus musculus GN=Ager PE=1 SV=2	GN=Ager	4.20
4	Q9JKR6	Hypoxia up-regulated protein 1 OS=Mus musculus GN=Hyou1 PE=1 SV=1	GN=Hyou1	3.62
5	P27786	Steroid 17-alpha-hydroxylase/17,20 lyase OS=Mus musculus GN=Cyp17a1 PE=1 SV=1	GN=Cyp17a1	3.32
6	P02089	Hemoglobin subunit beta-2 OS=Mus musculus GN=Hbb-b2 PE=1 SV=2	GN=Hbb-b2	3.16
7	P97425	Eosinophil cationic protein 2 OS=Mus musculus GN=Ear2 PE=2 SV=1	GN=Ear2	2.99
8	P97351	40S ribosomal protein S3a OS=Mus musculus GN=Rps3a PE=1 SV=3	GN=Rps3a	2.98
9	P31324	cAMP-dependent protein kinase type II-beta regulatory subunit OS=Mus musculus GN=Prkar2b PE=1 SV=3	GN=Prkar2b	2.87
10	P19324	Serpin H1 OS=Mus musculus GN=Serpinh1 PE=1 SV=3	GN=Serpinh1	2.73
11	E9Q718	Procollagen-lysine,2-oxoglutarate 5-dioxygenase 2 OS=Mus musculus GN=Plod2 PE=1 SV=1	GN=Plod2	2.57
12	Q9Z1T2	Thrombospondin-4 OS=Mus musculus GN=Thbs4 PE=1 SV=1	GN=Thbs4	2.52
13	P20029	78 kDa glucose-regulated protein OS=Mus musculus GN=Hspa5 PE=1 SV=3	GN=Hspa5	2.48
14	P51661	Corticosteroid 11-beta-dehydrogenase isozyme 2 OS=Mus musculus GN=Hsd11b2 PE=1 SV=2	GN=Hsd11b2	2.46
15	E9Q0U7	Heat shock protein 105 kDa OS=Mus musculus GN=Hsph1 PE=1 SV=1	GN=Hsph1	2.42
16	Q920L1	Fatty acid desaturase 1 OS=Mus musculus GN=Fads1 PE=1 SV=1	GN=Fads1	2.39
17	P09103	Protein disulfide-isomerase OS=Mus musculus GN=P4hb PE=1 SV=2	GN=P4hb	2.36
18	Q62010	Oviduct-specific glycoprotein OS=Mus musculus GN=Ovgp1 PE=2 SV=1	GN=Ovgp1	2.36
19	Q9DBS2	Tumor protein p63-regulated gene 1-like protein OS=Mus musculus GN=Tprg1l PE=1 SV=1	GN=Tprg1l	2.35
20	P11499	Heat shock protein HSP 90-beta OS=Mus musculus GN=Hsp90ab1 PE=1 SV=3	GN=Hsp90ab1	2.33
21	P63037	DnaJ homolog subfamily A member 1 OS=Mus musculus GN=Dnaja1 PE=1 SV=1	GN=Dnaja1	2.32
22	Q4FZE8	Major urinary protein 1 OS=Mus musculus GN=Mup1 PE=1 SV=1	GN=Mup1	2.29
23	A0A1D5RM85	60S ribosomal protein L18a (Fragment) OS=Mus musculus GN=Rpl18a PE=4 SV=1	GN=Rpl18a	2.27
24	Q3UU35	Ovostatin homolog OS=Mus musculus GN=Ovos PE=2 SV=2	GN=Ovos	2.26
25	Q9QZ82	Cholesterol side-chain cleavage enzyme, mitochondrial OS=Mus musculus GN=Cyp11a1 PE=1 SV=1	GN=Cyp11a1	2.24
26	Q8K2Z4	Condensin complex subunit 1 OS=Mus musculus GN=Ncapd2 PE=1 SV=2	GN=Ncapd2	2.24
27	Q920E5	Farnesyl pyrophosphate synthase OS=Mus musculus GN=Fdps PE=1 SV=1	GN=Fdps	2.17
28	P27659	60S ribosomal protein L3 OS=Mus musculus GN=Rpl3 PE=1 SV=3	GN=Rpl3	2.15
29	Q00898	Alpha-1-antitrypsin 1-5 OS=Mus musculus GN=Serpina1e PE=1 SV=1	GN=Serpina1e	2.12
30	F6VW30	14-3-3 protein theta (Fragment) OS=Mus musculus GN=Ywhaq PE=1 SV=1	GN=Ywhaq	2.12

31	E9QAZ2	Ribosomal protein L15 OS=Mus musculus GN=Gm10020 PE=3 SV=1	GN=Gm10020	2.11
32	P10126	Elongation factor 1-alpha 1 OS=Mus musculus GN=Eef1a1 PE=1 SV=3	GN=Eef1a1	2.11
33	A0A0A0MQA3	Alpha-1-antitrypsin 1-1 OS=Mus musculus GN=Serpina1a PE=1 SV=1	GN=Serpina1a	2.06
34	P62960	Nuclease-sensitive element-binding protein 1 OS=Mus musculus GN=Ybx1 PE=1 SV=3	GN=Ybx1	2.04
35	Q6NWW9	Fibronectin type III domain-containing protein 3B OS=Mus musculus GN=Fndc3b PE=1 SV=1	GN=Fndc3b	2.02
36	Q80ZP8	Armet protein OS=Mus musculus GN=Manf PE=1 SV=1	GN=Manf	2.02
37	Q3ULG5	DNA helicase OS=Mus musculus GN=Mcm6 PE=1 SV=1	GN=Mcm6	2.00
38	G3X8X6	Sialic acid binding Ig-like lectin 1, sialoadhesin, isoform CRA_b OS=Mus musculus GN=Siglec1 PE=1 SV=1	GN=Siglec1	1.99
39	A0A075B5P2	Protein Igkc (Fragment) OS=Mus musculus GN=Igkc PE=1 SV=1	GN=Igkc	1.98
40	B2RQC6	CAD protein OS=Mus musculus GN=Cad PE=1 SV=1	GN=Cad	1.97
41	P35980	60S ribosomal protein L18 OS=Mus musculus GN=Rpl18 PE=1 SV=3	GN=Rpl18	1.96
42	P62245	40S ribosomal protein S15a OS=Mus musculus GN=Rps15a PE=1 SV=2	GN=Rps15a	1.95
43	P68040	Receptor of activated protein C kinase 1 OS=Mus musculus GN=Rack1 PE=1 SV=3	GN=Rack1	1.95
44	Q04997	Inhibin alpha chain OS=Mus musculus GN=Inha PE=1 SV=2	GN=Inha	1.95
45	Q99LJ6	Glutathione peroxidase 7 OS=Mus musculus GN=Gpx7 PE=1 SV=1	GN=Gpx7	1.94
46	Q9JJF0	Nucleosome assembly protein 1-like 5 OS=Mus musculus GN=Nap1l5 PE=1 SV=1	GN=Nap1l5	1.93
47	Q9D8E6	60S ribosomal protein L4 OS=Mus musculus GN=Rpl4 PE=1 SV=3	GN=Rpl4	1.93
48	A0A140T8V5	Proliferating cell nuclear antigen OS=Mus musculus GN=Pcna-ps2 PE=3 SV=1	GN=Pcna-ps2	1.92
49	Q8BKC5	Importin-5 OS=Mus musculus GN=Ipo5 PE=1 SV=3	GN=Ipo5	1.91
50	P63276	40S ribosomal protein S17 OS=Mus musculus GN=Rps17 PE=1 SV=2	GN=Rps17	1.90
51	P08113	Endoplasmin OS=Mus musculus GN=Hsp90b1 PE=1 SV=2	GN=Hsp90b1	1.89
52	P47962	60S ribosomal protein L5 OS=Mus musculus GN=Rpl5 PE=1 SV=3	GN=Rpl5	1.89
53	P97926	Oxytocin receptor OS=Mus musculus GN=Oxtr PE=2 SV=2	GN=Oxtr	1.89
54	A2A690	Protein TANC2 OS=Mus musculus GN=Tanc2 PE=1 SV=1	GN=Tanc2	1.89
55	Q921X9	Protein disulfide-isomerase A5 OS=Mus musculus GN=Pdia5 PE=1 SV=1	GN=Pdia5	1.89
56	Q9Z0Z4	Hephaestin OS=Mus musculus GN=Heph PE=1 SV=3	GN=Heph	1.89
57	I7HLV2	60S ribosomal protein L10 (Fragment) OS=Mus musculus GN=Rpl10 PE=1 SV=1	GN=Rpl10	1.88
58	P25206	DNA replication licensing factor MCM3 OS=Mus musculus GN=Mcm3 PE=1 SV=2	GN=Mcm3	1.88
59	A6PW84	Prolyl 3-hydroxylase 1 OS=Mus musculus GN=P3h1 PE=1 SV=1	GN=P3h1	1.87
60	Q52KC3	DNA helicase OS=Mus musculus GN=Mcm5 PE=1 SV=1	GN=Mcm5	1.86
61	Q91W90	Thioredoxin domain-containing protein 5 OS=Mus musculus GN=Txndc5 PE=1 SV=2	GN=Txndc5	1.86
62	P14211	Calreticulin OS=Mus musculus GN=Calr PE=1 SV=1	GN=Calr	1.85
63	P57759	Endoplasmic reticulum resident protein 29 OS=Mus musculus GN=Erp29 PE=1 SV=2	GN=Erp29	1.85

64	P80316	T-complex protein 1 subunit epsilon OS=Mus musculus GN=Cct5 PE=1 SV=1	GN=Cct5	1.85
65	Q99PL5	Ribosome-binding protein 1 OS=Mus musculus GN=Rrbp1 PE=1 SV=2	GN=Rrbp1	1.84
66	P26645	Myristoylated alanine-rich C-kinase substrate OS=Mus musculus GN=Marcks PE=1 SV=2	GN=Marcks	1.84
67	Q6DFZ1	Golgi-specific brefeldin A-resistance factor 1 OS=Mus musculus GN=Gbf1 PE=1 SV=1	GN=Gbf1	1.84
68	P08905	Lysozyme C-2 OS=Mus musculus GN=Lyz2 PE=1 SV=2	GN=Lyz2	1.83
69	P62242	40S ribosomal protein S8 OS=Mus musculus GN=Rps8 PE=1 SV=2	GN=Rps8	1.83
70	A0A0A6YXQ0	Protein Ighv8-8 (Fragment) OS=Mus musculus GN=Ighv8-8 PE=4 SV=1	GN=Ighv8-8	1.83
71	Q9D8N0	Elongation factor 1-gamma OS=Mus musculus GN=Eef1g PE=1 SV=3	GN=Eef1g	1.83
72	Q8BVF2	Phosducin-like protein 3 OS=Mus musculus GN=Pdcl3 PE=1 SV=1	GN=Pdcl3	1.82
73	Q8K297	Procollagen galactosyltransferase 1 OS=Mus musculus GN=Colgalt1 PE=1 SV=2	GN=Colgalt1	1.80
74	F6V6T4	Transmembrane emp24 domain-containing protein 2 (Fragment) OS=Mus musculus GN=Tmed2 PE=1 SV=1	GN=Tmed2	1.80
75	P62827	GTP-binding nuclear protein Ran OS=Mus musculus GN=Ran PE=1 SV=3	GN=Ran	1.80
76	A0A0R4J0Z1	Protein disulfide-isomerase A4 OS=Mus musculus GN=Pdia4 PE=1 SV=1	GN=Pdia4	1.79
77	P34914	Bifunctional epoxide hydrolase 2 OS=Mus musculus GN=Ephx2 PE=1 SV=2	GN=Ephx2	1.79
78	E9PW66	Nucleosome assembly protein 1-like 1 OS=Mus musculus GN=Nap1l1 PE=1 SV=1	GN=Nap1l1	1.79
79	P14873	Microtubule-associated protein 1B OS=Mus musculus GN=Map1b PE=1 SV=2	GN=Map1b	1.78
80	Q61881	DNA replication licensing factor MCM7 OS=Mus musculus GN=Mcm7 PE=1 SV=1	GN=Mcm7	1.78
81	P07759	Serine protease inhibitor A3K OS=Mus musculus GN=Serpina3k PE=1 SV=2	GN=Serpina3k	1.78
82	P28649	Aromatase OS=Mus musculus GN=Cyp19a1 PE=2 SV=1	GN=Cyp19a1	1.78
83	P27773	Protein disulfide-isomerase A3 OS=Mus musculus GN=Pdia3 PE=1 SV=2	GN=Pdia3	1.77
84	Q3TIV5	Zinc finger CCCH domain-containing protein 15 OS=Mus musculus GN=Zc3h15 PE=1 SV=2	GN=Zc3h15	1.77
85	P58252	Elongation factor 2 OS=Mus musculus GN=Eef2 PE=1 SV=2	GN=Eef2	1.77
86	Q6VSS7	Protein Rhox8 OS=Mus musculus GN=Rhox8 PE=1 SV=1	GN=Rhox8	1.76
87	Q9CQ43	Deoxyuridine triphosphatase OS=Mus musculus GN=Dut PE=1 SV=1	GN=Dut	1.76
88	Q9D823	60S ribosomal protein L37 OS=Mus musculus GN=Rpl37 PE=3 SV=3	GN=Rpl37	1.76
89	Q8R2P8	Lysine--tRNA ligase OS=Mus musculus GN=Kars PE=1 SV=1	GN=Kars	1.75
90	P24369	Peptidyl-prolyl cis-trans isomerase B OS=Mus musculus GN=Ppib PE=1 SV=2	GN=Ppib	1.75
91	Q3UMP4	Plasminogen activator inhibitor 1 RNA-binding protein OS=Mus musculus GN=Serbp1 PE=1 SV=1	GN=Serbp1	1.74
92	P19253	60S ribosomal protein L13a OS=Mus musculus GN=Rpl13a PE=1 SV=4	GN=Rpl13a	1.74
93	P63017	Heat shock cognate 71 kDa protein OS=Mus musculus GN=Hspa8 PE=1 SV=1	GN=Hspa8	1.73
94	Q61553	Fascin OS=Mus musculus GN=Fscn1 PE=1 SV=4	GN=Fscn1	1.73
95	Q921Y0	MOB kinase activator 1A OS=Mus musculus GN=Mob1a PE=2 SV=3	GN=Mob1a	1.73
96	F6RPJ9	Insulin-degrading enzyme (Fragment) OS=Mus musculus GN=Ide PE=1 SV=1	GN=Ide	1.73

97	A2A547	Ribosomal protein L19 OS=Mus musculus GN=Rpl19 PE=1 SV=1	GN=Rpl19	1.72
98	B1ARA3	60S ribosomal protein L26 (Fragment) OS=Mus musculus GN=Rpl26 PE=1 SV=1	GN=Rpl26	1.72
99	Q8VDJ3	Vigilin OS=Mus musculus GN=Hdlbp PE=1 SV=1	GN=Hdlbp	1.71
100	Q8CDN6	Thioredoxin-like protein 1 OS=Mus musculus GN=Txnl1 PE=1 SV=3	GN=Txnl1	1.71
101	P14115	60S ribosomal protein L27a OS=Mus musculus GN=Rpl27a PE=1 SV=5	GN=Rpl27a	1.71
102	A0A1D5RLT6	GRAM domain-containing protein 1B OS=Mus musculus GN=Gramd1b PE=4 SV=1	GN=Gramd1b	1.69
103	Q8VCC9	Spondin-1 OS=Mus musculus GN=Spon1 PE=1 SV=1	GN=Spon1	1.69
104	P62270	40S ribosomal protein S18 OS=Mus musculus GN=Rps18 PE=1 SV=3	GN=Rps18	1.68
105	Q923L7	Ear6 protein OS=Mus musculus GN=Ear6 PE=1 SV=1	GN=Ear6	1.68
106	P04441	H-2 class II histocompatibility antigen gamma chain OS=Mus musculus GN=Cd74 PE=1 SV=3	GN=Cd74	1.67
107	Z4YJT3	La-related protein 1 OS=Mus musculus GN=Larp1 PE=1 SV=1	GN=Larp1	1.67
108	E9Q7B0	Prolyl 4-hydroxylase subunit alpha-1 OS=Mus musculus GN=P4ha1 PE=1 SV=1	GN=P4ha1	1.67
109	Q9DBX6	Cytochrome P450 2S1 OS=Mus musculus GN=Cyp2s1 PE=1 SV=1	GN=Cyp2s1	1.67
110	P61358	60S ribosomal protein L27 OS=Mus musculus GN=Rpl27 PE=1 SV=2	GN=Rpl27	1.66
111	E9QKZ2	Importin-9 OS=Mus musculus GN=Ipo9 PE=1 SV=1	GN=Ipo9	1.66
112	E9PZJ8	Activating signal cointegrator 1 complex subunit 3 OS=Mus musculus GN=Ascc3 PE=1 SV=1	GN=Ascc3	1.66
113	Q91WT7	3-alpha-hydroxysteroid dehydrogenase type 1 OS=Mus musculus GN=Akr1c14 PE=1 SV=1	GN=Akr1c14	1.66
114	Q78PY7	Staphylococcal nuclease domain-containing protein 1 OS=Mus musculus GN=Snd1 PE=1 SV=1	GN=Snd1	1.66
115	Q01853	Transitional endoplasmic reticulum ATPase OS=Mus musculus GN=Vcp PE=1 SV=4	GN=Vcp	1.66
116	Q99LE6	ATP-binding cassette sub-family F member 2 OS=Mus musculus GN=Abcf2 PE=1 SV=1	GN=Abcf2	1.66
117	Q8BMK4	Cytoskeleton-associated protein 4 OS=Mus musculus GN=Ckap4 PE=1 SV=2	GN=Ckap4	1.65
118	A2AGT5	Cytoskeleton-associated protein 5 OS=Mus musculus GN=Ckap5 PE=1 SV=1	GN=Ckap5	1.65
119	Q8CG48	Structural maintenance of chromosomes protein 2 OS=Mus musculus GN=Smc2 PE=1 SV=2	GN=Smc2	1.65
120	Q60865	Caprin-1 OS=Mus musculus GN=Caprin1 PE=1 SV=2	GN=Caprin1	1.65
121	Q9JIF7	Coatomer subunit beta OS=Mus musculus GN=Copb1 PE=1 SV=1	GN=Copb1	1.64
122	Q8BH76	DNA polymerase delta subunit 3 OS=Mus musculus GN=Pold3 PE=1 SV=1	GN=Pold3	1.64
123	Q3THK7	GMP synthase [glutamine-hydrolyzing] OS=Mus musculus GN=Gmps PE=1 SV=2	GN=Gmps	1.64
124	P80313	T-complex protein 1 subunit eta OS=Mus musculus GN=Cct7 PE=1 SV=1	GN=Cct7	1.64
125	Q9EPK6	Nucleotide exchange factor SIL1 OS=Mus musculus GN=Sil1 PE=1 SV=2	GN=Sil1	1.64
126	P24547	Inosine-5'-monophosphate dehydrogenase 2 OS=Mus musculus GN=Impdh2 PE=1 SV=2	GN=Impdh2	1.63
127	Q91W50	Cold shock domain-containing protein E1 OS=Mus musculus GN=Csde1 PE=1 SV=1	GN=Csde1	1.63
128	A0A0R4J170	Transcription activator BRG1 OS=Mus musculus GN=Smarca4 PE=1 SV=1	GN=Smarca4	1.63
129	B1AU75	Nuclear autoantigenic sperm protein OS=Mus musculus GN=Nasp PE=1 SV=1	GN=Nasp	1.63

130	Q8CG47	Structural maintenance of chromosomes protein 4 OS=Mus musculus GN=Smc4 PE=1 SV=1	GN=Smc4	1.62
131	Q5XJF6	Ribosomal protein OS=Mus musculus GN=Rpl10a PE=1 SV=1	GN=Rpl10a	1.62
132	P60229	Eukaryotic translation initiation factor 3 subunit E OS=Mus musculus GN=Eif3e PE=1 SV=1	GN=Eif3e	1.62
133	P62702	40S ribosomal protein S4, X isoform OS=Mus musculus GN=Rps4x PE=1 SV=2	GN=Rps4x	1.62
134	O88569	Heterogeneous nuclear ribonucleoproteins A2/B1 OS=Mus musculus GN=Hnrnpa2b1 PE=1 SV=2	GN=Hnrnpa2b1	1.61
135	P97310	DNA replication licensing factor MCM2 OS=Mus musculus GN=Mcm2 PE=1 SV=3	GN=Mcm2	1.61
136	Q6ZWN5	40S ribosomal protein S9 OS=Mus musculus GN=Rps9 PE=1 SV=3	GN=Rps9	1.61
137	Q3UNN4	SWI/SNF complex subunit SMARCC1 OS=Mus musculus GN=Smarcc1 PE=1 SV=1	GN=Smarcc1	1.61
138	O35685	Nuclear migration protein nudC OS=Mus musculus GN=Nudc PE=1 SV=1	GN=Nudc	1.60
139	P60710	Actin, cytoplasmic 1 OS=Mus musculus GN=Actb PE=1 SV=1	GN=Actb	1.60
140	P05201	Aspartate aminotransferase, cytoplasmic OS=Mus musculus GN=Got1 PE=1 SV=3	GN=Got1	1.60
141	P07901	Heat shock protein HSP 90-alpha OS=Mus musculus GN=Hsp90aa1 PE=1 SV=4	GN=Hsp90aa1	1.60
142	Q60864	Stress-induced-phosphoprotein 1 OS=Mus musculus GN=Stip1 PE=1 SV=1	GN=Stip1	1.60
143	Q91YW3	DnaJ homolog subfamily C member 3 OS=Mus musculus GN=Dnajc3 PE=1 SV=1	GN=Dnajc3	1.60
144	A0A075B666	Protein Igkv13-85 (Fragment) OS=Mus musculus GN=Igkv13-85 PE=4 SV=5	GN=Igkv13-85	1.60
145	Q8CGC7	Bifunctional glutamate/proline--tRNA ligase OS=Mus musculus GN=Eprs PE=1 SV=4	GN=Eprs	1.59
146	Q9WVL3	Solute carrier family 12 member 7 OS=Mus musculus GN=Slc12a7 PE=1 SV=1	GN=Slc12a7	1.58
147	P14869	60S acidic ribosomal protein P0 OS=Mus musculus GN=Rplp0 PE=1 SV=3	GN=Rplp0	1.58
148	Q08EB6	CLIP associating protein 2 OS=Mus musculus GN=Clasp2 PE=1 SV=1	GN=Clasp2	1.58
149	Q04750	DNA topoisomerase 1 OS=Mus musculus GN=Top1 PE=1 SV=2	GN=Top1	1.58
150	Q6PB66	Leucine-rich PPR motif-containing protein, mitochondrial OS=Mus musculus GN=Lrpprc PE=1 SV=2	GN=Lrpprc	1.57
151	Q3TML0	Protein disulfide-isomerase A6 OS=Mus musculus GN=Pdia6 PE=1 SV=1	GN=Pdia6	1.57
152	P49722	Proteasome subunit alpha type-2 OS=Mus musculus GN=Psma2 PE=1 SV=3	GN=Psma2	1.56
153	Q61576	Peptidyl-prolyl cis-trans isomerase FKBP10 OS=Mus musculus GN=Fkbp10 PE=1 SV=2	GN=Fkbp10	1.56
154	E9PZF0	Nucleoside diphosphate kinase OS=Mus musculus GN=Gm20390 PE=3 SV=1	GN=Gm20390	1.56
155	P12970	60S ribosomal protein L7a OS=Mus musculus GN=Rpl7a PE=1 SV=2	GN=Rpl7a	1.55
156	Q99M31	Heat shock 70 kDa protein 14 OS=Mus musculus GN=Hspa14 PE=1 SV=2	GN=Hspa14	1.55
157	Q9CZX8	40S ribosomal protein S19 OS=Mus musculus GN=Rps19 PE=1 SV=3	GN=Rps19	1.55
158	F6TQW2	Protein Ighg2c OS=Mus musculus GN=Ighg2c PE=1 SV=1	GN=Ighg2c	1.54
159	P30416	Peptidyl-prolyl cis-trans isomerase FKBP4 OS=Mus musculus GN=Fkbp4 PE=1 SV=5	GN=Fkbp4	1.54
160	P57776	Elongation factor 1-delta OS=Mus musculus GN=Eef1d PE=1 SV=3	GN=Eef1d	1.54
161	P56379	6.8 kDa mitochondrial proteolipid OS=Mus musculus GN=Mp68 PE=1 SV=1	GN=Mp68	1.54
162	Q6ZWZ4	60S ribosomal protein L36 OS=Mus musculus GN=Rpl36 PE=1 SV=1	GN=Rpl36	1.53

163	Q8R050	Eukaryotic peptide chain release factor GTP-binding subunit ERF3A OS=Mus musculus GN=Gspt1 PE=1 SV=2	GN=Gspt1	1.53
164	P63038	60 kDa heat shock protein, mitochondrial OS=Mus musculus GN=Hspd1 PE=1 SV=1	GN=Hspd1	1.53
165	D3Z0F5	COP9 signalosome complex subunit 6 OS=Mus musculus GN=Cops6 PE=1 SV=1	GN=Cops6	1.52
166	Q9QXS6	Drebrin OS=Mus musculus GN=Dbn1 PE=1 SV=4	GN=Dbn1	1.52
167	P14148	60S ribosomal protein L7 OS=Mus musculus GN=Rpl7 PE=1 SV=2	GN=Rpl7	1.52
168	P35564	Calnexin OS=Mus musculus GN=Canx PE=1 SV=1	GN=Canx	1.52
169	Q6PGH2	Hematological and neurological expressed 1-like protein OS=Mus musculus GN=Hn1l PE=1 SV=1	GN=Hn1l	1.51
170	Q80YX1	Tenascin OS=Mus musculus GN=Tnc PE=1 SV=1	GN=Tnc	1.51
171	E9Q035	Protein Gm20425 OS=Mus musculus GN=Gm20425 PE=4 SV=1	GN=Gm20425	1.51
172	D3Z136	Elongation of very long chain fatty acids protein (Fragment) OS=Mus musculus GN=Elovl5 PE=1 SV=1	GN=Elovl5	1.51
173	Q6PHQ8	N-alpha-acetyltransferase 35, NatC auxiliary subunit OS=Mus musculus GN=Naa35 PE=1 SV=1	GN=Naa35	1.50
174	Q9ERK4	Exportin-2 OS=Mus musculus GN=Cse1l PE=1 SV=1	GN=Cse1l	1.50
175	Q9Z1Z0	General vesicular transport factor p115 OS=Mus musculus GN=Uso1 PE=1 SV=2	GN=Uso1	1.50
176	Q99020	Heterogeneous nuclear ribonucleoprotein A/B OS=Mus musculus GN=Hnrnpab PE=1 SV=1	GN=Hnrnpab	1.50
177	Q05D44	Eukaryotic translation initiation factor 5B OS=Mus musculus GN=Eif5b PE=1 SV=2	GN=Eif5b	1.50
178	A0A140T8T4	Protein Rpl9-ps6 OS=Mus musculus GN=Rpl9-ps6 PE=4 SV=1	GN=Rpl9-ps6	1.49
179	Q9CQN1	Heat shock protein 75 kDa, mitochondrial OS=Mus musculus GN=Trap1 PE=1 SV=1	GN=Trap1	1.49
180	Q3TUE1	Far upstream element-binding protein 1 OS=Mus musculus GN=Fubp1 PE=1 SV=1	GN=Fubp1	1.49

up-regulated 1.5-fold proteins

N	Citable Accession	Name	Gene Name	Fold Change
1	Q3U7R1	Extended synaptotagmin-1 OS=Mus musculus GN=Esyt1 PE=1 SV=2	GN=Esyt1	1.51
2	E9QJW9	Tryptase beta-2 OS=Mus musculus GN=Tpsb2 PE=1 SV=1	GN=Tpsb2	1.51
3	Q9D051	Pyruvate dehydrogenase E1 component subunit beta, mitochondrial OS=Mus musculus GN=Pdhb PE=1 SV=1	GN=Pdhb	1.51
4	Q3ULJ0	Glycerol-3-phosphate dehydrogenase 1-like protein OS=Mus musculus GN=Gpd1l PE=1 SV=2	GN=Gpd1l	1.51
5	A0A0R4J0P1	Acyl-Coenzyme A dehydrogenase family, member 8 OS=Mus musculus GN=Acad8 PE=1 SV=1	GN=Acad8	1.51
6	A0A0J9YUZ4	High mobility group protein B1 (Fragment) OS=Mus musculus GN=Hmgb1 PE=1 SV=1	GN=Hmgb1	1.52
7	E9QP62	LIM and senescent cell antigen-like-containing domain protein 1 OS=Mus musculus GN=Lims1 PE=1 SV=1	GN=Lims1	1.52
8	O70318	Band 4.1-like protein 2 OS=Mus musculus GN=Epb41l2 PE=1 SV=2	GN=Epb41l2	1.52
9	Q8CFB8	Poly [ADP-ribose] polymerase OS=Mus musculus GN=Parp3 PE=1 SV=1	GN=Parp3	1.52
10	P14733	Lamin-B1 OS=Mus musculus GN=Lmnb1 PE=1 SV=3	GN=Lmnb1	1.52
11	Q9QZQ8	Core histone macro-H2A.1 OS=Mus musculus GN=H2afy PE=1 SV=3	GN=H2afy	1.53
12	A0A140T8W1	Collagen alpha-5(VI) chain OS=Mus musculus GN=Col6a5 PE=1 SV=1	GN=Col6a5	1.54

13	Q01339	Beta-2-glycoprotein 1 OS=Mus musculus GN=Apoh PE=1 SV=1	GN=Apoh	1.54
14	P14602	Heat shock protein beta-1 OS=Mus musculus GN=Hspb1 PE=1 SV=3	GN=Hspb1	1.54
15	Q91XH5	Sepiapterin reductase OS=Mus musculus GN=Spr PE=1 SV=1	GN=Spr	1.55
16	A2AJY2	Collagen alpha-1(XV) chain OS=Mus musculus GN=Col15a1 PE=1 SV=1	GN=Col15a1	1.55
17	J3QQ16	Protein Col6a3 OS=Mus musculus GN=Col6a3 PE=1 SV=1	GN=Col6a3	1.56
18	Q99JA0	Calcitonin gene-related peptide 1 OS=Mus musculus GN=Calca PE=2 SV=1	GN=Calca	1.56
19	Q91Y97	Fructose-bisphosphate aldolase B OS=Mus musculus GN=Aldob PE=1 SV=3	GN=Aldob	1.57
20	P08226	Apolipoprotein E OS=Mus musculus GN=Apoe PE=1 SV=2	GN=Apoe	1.57
21	P10833	Ras-related protein R-Ras OS=Mus musculus GN=Rras PE=1 SV=1	GN=Rras	1.58
22	Q9R0P9	Ubiquitin carboxyl-terminal hydrolase isozyme L1 OS=Mus musculus GN=Uchl1 PE=1 SV=1	GN=Uchl1	1.58
23	Q5SYD0	Unconventional myosin-Id OS=Mus musculus GN=Myo1d PE=1 SV=1	GN=Myo1d	1.58
24	Q8BFQ1	Prosaposin OS=Mus musculus GN=Psap PE=1 SV=1	GN=Psap	1.58
25	A2AQZ7	Phytanoyl-CoA dioxygenase domain-containing protein 1 OS=Mus musculus GN=Phyhd1 PE=1 SV=1	GN=Phyhd1	1.58
26	P20918	Plasminogen OS=Mus musculus GN=Plg PE=1 SV=3	GN=Plg	1.59
27	P32020	Non-specific lipid-transfer protein OS=Mus musculus GN=Scp2 PE=1 SV=3	GN=Scp2	1.59
28	P19001	Keratin, type I cytoskeletal 19 OS=Mus musculus GN=Krt19 PE=1 SV=1	GN=Krt19	1.59
29	Q99K41	EMILIN-1 OS=Mus musculus GN=Emilin1 PE=1 SV=1	GN=Emilin1	1.60
30	P84228	Histone H3.2 OS=Mus musculus GN=Hist1h3b PE=1 SV=2	GN=Hist1h3b	1.61
31	Q99MZ7	Peroxisomal trans-2-enoyl-CoA reductase OS=Mus musculus GN=Pecr PE=1 SV=1	GN=Pecr	1.61
32	P11531	Dystrophin OS=Mus musculus GN=Dmd PE=1 SV=3	GN=Dmd	1.62
33	Q91V76	Ester hydrolase C11orf54 homolog OS=Mus musculus PE=1 SV=1	GN=--	1.62
34	Q8K0T0	Reticulon-1 OS=Mus musculus GN=Rtn1 PE=1 SV=1	GN=Rtn1	1.62
35	X1WI18	CMRF35-like molecule 9 (Fragment) OS=Mus musculus GN=Cd300lg PE=1 SV=7	GN=Cd300lg	1.62
36	Q9WVK4	EH domain-containing protein 1 OS=Mus musculus GN=Ehd1 PE=1 SV=1	GN=Ehd1	1.62
37	P13020	Gelsolin OS=Mus musculus GN=Gsn PE=1 SV=3	GN=Gsn	1.62
38	P14142	Solute carrier family 2, facilitated glucose transporter member 4 OS=Mus musculus GN=Slc2a4 PE=1 SV=3	GN=Slc2a4	1.62
39	E9Q616	Protein Ahnak OS=Mus musculus GN=Ahnak PE=1 SV=1	GN=Ahnak	1.62
40	Q8BFW7	Lipoma-preferred partner homolog OS=Mus musculus GN=Lpp PE=1 SV=1	GN=Lpp	1.62
41	E9PV48	Protein Ifit3b OS=Mus musculus GN=Ifit3b PE=1 SV=1	GN=Ifit3b	1.62
42	Q61001	Laminin subunit alpha-5 OS=Mus musculus GN=Lama5 PE=1 SV=4	GN=Lama5	1.63
43	A0A0R4J0I6	Acyl-CoA dehydrogenase family member 11 OS=Mus musculus GN=Acad11 PE=1 SV=1	GN=Acad11	1.63
44	Q9Z2V4	Phosphoenolpyruvate carboxykinase, cytosolic [GTP] OS=Mus musculus GN=Pck1 PE=1 SV=1	GN=Pck1	1.63
45	Q61735	Leukocyte surface antigen CD47 OS=Mus musculus GN=Cd47 PE=1 SV=2	GN=Cd47	1.64

46	Q9Z0K8	Pantetheinase OS=Mus musculus GN=Vnn1 PE=1 SV=3	GN=Vnn1	1.64
47	E9Q6R7	Protein Utrn OS=Mus musculus GN=Utrn PE=1 SV=1	GN=Utrn	1.64
48	G3X8Q5	Ceruloplasmin OS=Mus musculus GN=Cp PE=1 SV=1	GN=Cp	1.65
49	Q07797	Galectin-3-binding protein OS=Mus musculus GN=Lgals3bp PE=1 SV=1	GN=Lgals3bp	1.65
50	Q8CGN5	Perilipin-1 OS=Mus musculus GN=Plin1 PE=1 SV=2	GN=Plin1	1.65
51	Q8VDF2	E3 ubiquitin-protein ligase UHRF1 OS=Mus musculus GN=Uhrf1 PE=1 SV=2	GN=Uhrf1	1.66
52	M0QWP1	Agrin OS=Mus musculus GN=Agrn PE=1 SV=1	GN=Agrn	1.66
53	P21956	Lactadherin OS=Mus musculus GN=Mfge8 PE=1 SV=3	GN=Mfge8	1.66
54	P18872	Guanine nucleotide-binding protein G(o) subunit alpha OS=Mus musculus GN=Gnao1 PE=1 SV=3	GN=Gnao1	1.67
55	Q8CCK0	Core histone macro-H2A.2 OS=Mus musculus GN=H2afy2 PE=1 SV=3	GN=H2afy2	1.67
56	A0A0R4J083	Long-chain-specific acyl-CoA dehydrogenase, mitochondrial OS=Mus musculus GN=Acadl PE=1 SV=1	GN=Acadl	1.67
57	Q8BX02	KN motif and ankyrin repeat domain-containing protein 2 OS=Mus musculus GN=Kank2 PE=1 SV=1	GN=Kank2	1.68
58	A2A7A7	GDH/6PGL endoplasmic bifunctional protein OS=Mus musculus GN=H6pd PE=1 SV=1	GN=H6pd	1.68
59	P08207	Protein S100-A10 OS=Mus musculus GN=S100a10 PE=1 SV=2	GN=S100a10	1.69
60	Q4VWZ5	Acyl-CoA-binding protein OS=Mus musculus GN=Dbi PE=1 SV=1	GN=Dbi	1.69
61	Q9R269	Periplakin OS=Mus musculus GN=Ppl PE=1 SV=1	GN=Ppl	1.69
62	P14152	Malate dehydrogenase, cytoplasmic OS=Mus musculus GN=Mdh1 PE=1 SV=3	GN=Mdh1	1.69
63	P35505	Fumarylacetoacetase OS=Mus musculus GN=Fah PE=1 SV=2	GN=Fah	1.69
64	E9PWQ3	Protein Col6a3 OS=Mus musculus GN=Col6a3 PE=1 SV=2	GN=Col6a3	1.69
65	P16125	L-lactate dehydrogenase B chain OS=Mus musculus GN=Ldhb PE=1 SV=2	GN=Ldhb	1.70
66	D3Z080	Sorbin and SH3 domain-containing protein 2 OS=Mus musculus GN=Sorbs2 PE=1 SV=1	GN=Sorbs2	1.70
67	Q7TNG8	Probable D-lactate dehydrogenase, mitochondrial OS=Mus musculus GN=Ldhd PE=1 SV=1	GN=Ldhd	1.70
68	Q9R0H0	Peroxisomal acyl-coenzyme A oxidase 1 OS=Mus musculus GN=Acox1 PE=1 SV=5	GN=Acox1	1.70
69	E9QN70	Laminin subunit beta-1 OS=Mus musculus GN=Lamb1 PE=1 SV=1	GN=Lamb1	1.72
70	Q8R0Y6	Cytosolic 10-formyltetrahydrofolate dehydrogenase OS=Mus musculus GN=Aldh1l1 PE=1 SV=1	GN=Aldh1l1	1.72
71	Q9CRB1	Galectin OS=Mus musculus GN=Lgals7 PE=1 SV=1	GN=Lgals7	1.72
72	P10649	Glutathione S-transferase Mu 1 OS=Mus musculus GN=Gstm1 PE=1 SV=2	GN=Gstm1	1.73
73	Q8K2I3	Dimethylaniline monooxygenase [N-oxide-forming] 2 OS=Mus musculus GN=Fmo2 PE=1 SV=3	GN=Fmo2	1.73
74	E9PUM4	Talin-2 OS=Mus musculus GN=Tln2 PE=1 SV=1	GN=Tln2	1.73
75	Q8VHY0	Chondroitin sulfate proteoglycan 4 OS=Mus musculus GN=Cspg4 PE=1 SV=3	GN=Cspg4	1.73
76	Q9QXC1	Fetuin-B OS=Mus musculus GN=Fetub PE=1 SV=1	GN=Fetub	1.74
77	Q9D1H9	Microfibril-associated glycoprotein 4 OS=Mus musculus GN=Mfap4 PE=1 SV=1	GN=Mfap4	1.74
78	P33267	Cytochrome P450 2F2 OS=Mus musculus GN=Cyp2f2 PE=1 SV=1	GN=Cyp2f2	1.74

79	P40936	Indolethylamine N-methyltransferase OS=Mus musculus GN=Inmt PE=1 SV=1	GN=Inmt	1.74
80	P11679	Keratin, type II cytoskeletal 8 OS=Mus musculus GN=Krt8 PE=1 SV=4	GN=Krt8	1.75
81	P11404	Fatty acid-binding protein, heart OS=Mus musculus GN=Fabp3 PE=1 SV=5	GN=Fabp3	1.76
82	P10493	Nidogen-1 OS=Mus musculus GN=Nid1 PE=1 SV=2	GN=Nid1	1.76
83	Q9CZS1	Aldehyde dehydrogenase X, mitochondrial OS=Mus musculus GN=Aldh1b1 PE=1 SV=1	GN=Aldh1b1	1.77
84	O35682	Myeloid-associated differentiation marker OS=Mus musculus GN=Myadm PE=1 SV=2	GN=Myadm	1.77
85	E9Q3B9	Monoglyceride lipase OS=Mus musculus GN=Mgll PE=1 SV=1	GN=Mgll	1.77
86	Q03734	Serine protease inhibitor A3M OS=Mus musculus GN=Serpina3m PE=1 SV=2	GN=Serpina3m	1.77
87	Q64282	Interferon-induced protein with tetratricopeptide repeats 1 OS=Mus musculus GN=Ifit1 PE=1 SV=2	GN=Ifit1	1.78
88	Q9D9V3	Ethylmalonyl-CoA decarboxylase OS=Mus musculus GN=Echdc1 PE=1 SV=2	GN=Echdc1	1.79
89	Q8BX70	Vacuolar protein sorting-associated protein 13C OS=Mus musculus GN=Vps13c PE=1 SV=2	GN=Vps13c	1.80
90	Q8BW75	Amine oxidase [flavin-containing] B OS=Mus musculus GN=Maob PE=1 SV=4	GN=Maob	1.80
91	P54869	Hydroxymethylglutaryl-CoA synthase, mitochondrial OS=Mus musculus GN=Hmgcs2 PE=1 SV=2	GN=Hmgcs2	1.80
92	Q91VJ2	Protein kinase C delta-binding protein OS=Mus musculus GN=Prkcdbp PE=1 SV=1	GN=Prkcdbp	1.80
93	P43883	Perilipin-2 OS=Mus musculus GN=Plin2 PE=1 SV=2	GN=Plin2	1.80
94	F8WIX8	Histone H2A OS=Mus musculus GN=Hist1h2al PE=3 SV=1	GN=Hist1h2al	1.80
95	P20152	Vimentin OS=Mus musculus GN=Vim PE=1 SV=3	GN=Vim	1.80
96	B8JK39	Integrin alpha-9 OS=Mus musculus GN=Itga9 PE=1 SV=1	GN=Itga9	1.80
97	P10648	Glutathione S-transferase A2 OS=Mus musculus GN=Gsta2 PE=1 SV=3	GN=Gsta2	1.80
98	Q9Z2A9	Gamma-glutamyltransferase 5 OS=Mus musculus GN=Ggt5 PE=1 SV=2	GN=Ggt5	1.81
99	E9Q0S6	Protein Tns1 OS=Mus musculus GN=Tns1 PE=1 SV=1	GN=Tns1	1.82
100	Q64727	Vinculin OS=Mus musculus GN=Vcl PE=1 SV=4	GN=Vcl	1.83
101	Q08091	Calponin-1 OS=Mus musculus GN=Cnn1 PE=1 SV=1	GN=Cnn1	1.83
102	E9QNA7	Sorbin and SH3 domain-containing protein 1 OS=Mus musculus GN=Sorbs1 PE=1 SV=1	GN=Sorbs1	1.84
103	Q9R1M5	NACHT, LRR and PYD domains-containing protein 5 OS=Mus musculus GN=Nlrp5 PE=1 SV=2	GN=Nlrp5	1.84
104	Q9Z2D6	Methyl-CpG-binding protein 2 OS=Mus musculus GN=Mecp2 PE=1 SV=1	GN=Mecp2	1.85
105	Q9R111	Guanine deaminase OS=Mus musculus GN=Gda PE=1 SV=1	GN=Gda	1.85
106	P58242	Acid sphingomyelinase-like phosphodiesterase 3b OS=Mus musculus GN=Smpdl3b PE=1 SV=1	GN=Smpdl3b	1.86
107	P62806	Histone H4 OS=Mus musculus GN=Hist1h4a PE=1 SV=2	GN=Hist1h4a	1.86
108	Q62009	Periostin OS=Mus musculus GN=Postn PE=1 SV=2	GN=Postn	1.88
109	E9PZ16	Basement membrane-specific heparan sulfate proteoglycan core protein OS=Mus musculus GN=Hspg2 PE=1 SV=1	GN=Hspg2	1.88
110	P00920	Carbonic anhydrase 2 OS=Mus musculus GN=Ca2 PE=1 SV=4	GN=Ca2	1.88
111	P28843	Dipeptidyl peptidase 4 OS=Mus musculus GN=Dpp4 PE=1 SV=3	GN=Dpp4	1.89

112	P51910	Apolipoprotein D OS=Mus musculus GN=Apod PE=1 SV=1	GN=Apod	1.93
113	B7ZN28	Papilin OS=Mus musculus GN=Papln PE=1 SV=1	GN=Papln	1.93
114	Q60847	Collagen alpha-1(XII) chain OS=Mus musculus GN=Col12a1 PE=2 SV=3	GN=Col12a1	1.93
115	G3X8X3	Large neutral amino acids transporter small subunit 3 OS=Mus musculus GN=Slc43a1 PE=1 SV=1	GN=Slc43a1	1.93
116	Q9ESU7	Amino acid transporter OS=Mus musculus GN=Slc1a5 PE=1 SV=1	GN=Slc1a5	1.94
117	Q9WVB3	Transducin-like enhancer protein 6 OS=Mus musculus GN=Tle6 PE=1 SV=1	GN=Tle6	1.95
118	Q62005	Zona pellucida sperm-binding protein 1 OS=Mus musculus GN=Zp1 PE=1 SV=1	GN=Zp1	1.95
119	Q9WTI7	Unconventional myosin-Ic OS=Mus musculus GN=Myo1c PE=1 SV=2	GN=Myo1c	1.98
120	P10922	Histone H1.0 OS=Mus musculus GN=H1f0 PE=2 SV=4	GN=H1f0	2.00
121	P97449	Aminopeptidase N OS=Mus musculus GN=Anpep PE=1 SV=4	GN=Anpep	2.00
122	Q9D997	N-acetyl-D-glucosamine kinase OS=Mus musculus GN=Nagk PE=1 SV=1	GN=Nagk	2.01
123	P46412	Glutathione peroxidase 3 OS=Mus musculus GN=Gpx3 PE=1 SV=2	GN=Gpx3	2.01
124	Q8CGB6	Tensin-2 OS=Mus musculus GN=Tns2 PE=1 SV=1	GN=Tns2	2.01
125	Q6B966	NACHT, LRR and PYD domains-containing protein 14 OS=Mus musculus GN=Nlrp14 PE=2 SV=2	GN=Nlrp14	2.01
126	Q61292	Laminin subunit beta-2 OS=Mus musculus GN=Lamb2 PE=1 SV=2	GN=Lamb2	2.02
127	Q9DBM2	Peroxisomal bifunctional enzyme OS=Mus musculus GN=Ehhadh PE=1 SV=4	GN=Ehhadh	2.03
128	Q9WVL0	Maleylacetoacetate isomerase OS=Mus musculus GN=Gstz1 PE=1 SV=1	GN=Gstz1	2.04
129	Q64471	Glutathione S-transferase theta-1 OS=Mus musculus GN=Gstt1 PE=1 SV=4	GN=Gstt1	2.04
130	Q60675	Laminin subunit alpha-2 OS=Mus musculus GN=Lama2 PE=1 SV=2	GN=Lama2	2.04
131	Q64475	Histone H2B type 1-B OS=Mus musculus GN=Hist1h2bb PE=1 SV=3	GN=Hist1h2bb	2.05
132	Q9WVH9	Fibulin-5 OS=Mus musculus GN=Fbln5 PE=1 SV=1	GN=Fbln5	2.05
133	P06728	Apolipoprotein A-IV OS=Mus musculus GN=Apoa4 PE=1 SV=3	GN=Apoa4	2.06
134	P21619	Lamin-B2 OS=Mus musculus GN=Lmnb2 PE=1 SV=2	GN=Lmnb2	2.06
135	Q64GA5	Egg and early embryo abundant protein OS=Mus musculus GN=Pla2g4c PE=1 SV=1	GN=Pla2g4c	2.08
136	P97315	Cysteine and glycine-rich protein 1 OS=Mus musculus GN=Csrp1 PE=1 SV=3	GN=Csrp1	2.08
137	G3X8T9	Serine (Or cysteine) peptidase inhibitor, clade A, member 3N, isoform CRA_a OS=Mus musculus GN=Serpina3n PE=1 SV=1	GN=Serpina3n	2.09
138	O35452	Protein Tnxb OS=Mus musculus GN=Tnxb PE=1 SV=1	GN=Tnxb	2.11
139	A0A087WRE7	Hydroxyacid-oxoacid transhydrogenase, mitochondrial (Fragment) OS=Mus musculus GN=Adhfe1 PE=1 SV=1	GN=Adhfe1	2.11
140	Q91YR9	Prostaglandin reductase 1 OS=Mus musculus GN=Ptgr1 PE=1 SV=2	GN=Ptgr1	2.12
141	F8VQJ3	Laminin subunit gamma-1 OS=Mus musculus GN=Lamc1 PE=1 SV=1	GN=Lamc1	2.14
142	Q00519	Xanthine dehydrogenase/oxidase OS=Mus musculus GN=Xdh PE=1 SV=5	GN=Xdh	2.14
143	P82347	Delta-sarcoglycan OS=Mus musculus GN=Sgcd PE=1 SV=1	GN=Sgcd	2.16

144	L7N1W9	NACHT, LRR and PYD domains-containing protein 4F OS=Mus musculus GN=Nlrp4f PE=4 SV=1	GN=Nlrp4f	2.16
145	G5E8R3	Pyruvate carboxylase OS=Mus musculus GN=Pcx PE=1 SV=1	GN=Pcx	2.19
146	Q9WTR5	Cadherin-13 OS=Mus musculus GN=Cdh13 PE=1 SV=2	GN=Cdh13	2.19
147	P17563	Selenium-binding protein 1 OS=Mus musculus GN=Selenbp1 PE=1 SV=2	GN=Selenbp1	2.19
148	O09131	Glutathione S-transferase omega-1 OS=Mus musculus GN=Gsto1 PE=1 SV=2	GN=Gsto1	2.20
149	P48678	Prelamin-A/C OS=Mus musculus GN=Lmna PE=1 SV=2	GN=Lmna	2.21
150	A8DUK4	Beta-globin OS=Mus musculus GN=Hbbt1 PE=1 SV=1	GN=Hbbt1	2.22
151	P08122	Collagen alpha-2(IV) chain OS=Mus musculus GN=Col4a2 PE=1 SV=4	GN=Col4a2	2.23
152	Q8K3V4	Protein-arginine deiminase type-6 OS=Mus musculus GN=Padi6 PE=1 SV=2	GN=Padi6	2.23
153	Q9JK53	Prolargin OS=Mus musculus GN=Prelp PE=1 SV=2	GN=Prelp	2.23
154	Q6NSP9	High mobility group protein HMGI-C OS=Mus musculus GN=Hmga2 PE=1 SV=1	GN=Hmga2	2.25
155	Q8JZU2	Tricarboxylate transport protein, mitochondrial OS=Mus musculus GN=Slc25a1 PE=1 SV=1	GN=Slc25a1	2.25
156	P97927	Laminin subunit alpha-4 OS=Mus musculus GN=Lama4 PE=1 SV=2	GN=Lama4	2.25
157	O88322	Nidogen-2 OS=Mus musculus GN=Nid2 PE=1 SV=2	GN=Nid2	2.25
158	O70423	Membrane primary amine oxidase OS=Mus musculus GN=Aoc3 PE=1 SV=3	GN=Aoc3	2.27
159	Q9Z0F7	Gamma-synuclein OS=Mus musculus GN=Sncg PE=1 SV=1	GN=Sncg	2.27
160	Q61738	Integrin alpha-7 OS=Mus musculus GN=Itga7 PE=1 SV=3	GN=Itga7	2.28
161	P50285	Dimethylaniline monooxygenase [N-oxide-forming] 1 OS=Mus musculus GN=Fmo1 PE=1 SV=1	GN=Fmo1	2.30
162	Q9EQ20	Methylmalonate-semialdehyde dehydrogenase [acylating], mitochondrial OS=Mus musculus GN=Aldh6a1 PE=1 SV=1	GN=Aldh6a1	2.31
163	B7ZNH7	Collagen alpha-1(XIV) chain OS=Mus musculus GN=Col14a1 PE=1 SV=1	GN=Col14a1	2.32
164	P09528	Ferritin heavy chain OS=Mus musculus GN=Fth1 PE=1 SV=2	GN=Fth1	2.33
165	P16045	Galectin-1 OS=Mus musculus GN=Lgals1 PE=1 SV=3	GN=Lgals1	2.34
166	P24270	Catalase OS=Mus musculus GN=Cat PE=1 SV=4	GN=Cat	2.40
167	Q61554	Fibrillin-1 OS=Mus musculus GN=Fbn1 PE=1 SV=2	GN=Fbn1	2.41
168	P47738	Aldehyde dehydrogenase, mitochondrial OS=Mus musculus GN=Aldh2 PE=1 SV=1	GN=Aldh2	2.42
169	Q91W19	Sulfotransferase OS=Mus musculus GN=Sult1a1 PE=1 SV=1	GN=Sult1a1	2.44
170	Q9CWU5	KH domain-containing protein 3 OS=Mus musculus GN=Khdc3 PE=1 SV=1	GN=Khdc3	2.45
171	Q9CPX4	Ferritin OS=Mus musculus GN=Ftl1-ps1 PE=1 SV=1	GN=Ftl1-ps1	2.48
172	P28654	Decorin OS=Mus musculus GN=Dcn PE=1 SV=1	GN=Dcn	2.51
173	Q8C6K9	Collagen alpha-6(VI) chain OS=Mus musculus GN=Col6a6 PE=1 SV=2	GN=Col6a6	2.54
174	Q8C253	Galectin OS=Mus musculus GN=Lgals3 PE=1 SV=1	GN=Lgals3	2.54
175	Q3THW5	Histone H2A.V OS=Mus musculus GN=H2afv PE=1 SV=3	GN=H2afv	2.63
176	P07356	Annexin A2 OS=Mus musculus GN=Anxa2 PE=1 SV=2	GN=Anxa2	2.64

177	E9Q4M2	Hormone-sensitive lipase OS=Mus musculus GN=Lipe PE=1 SV=1	GN=Lipe	2.66
178	Q6IFX2	Keratin, type I cytoskeletal 42 OS=Mus musculus GN=Krt42 PE=1 SV=1	GN=Krt42	2.67
179	O54724	Polymerase I and transcript release factor OS=Mus musculus GN=Ptrf PE=1 SV=1	GN=Ptrf	2.67
180	Q8BJ64	Choline dehydrogenase, mitochondrial OS=Mus musculus GN=Chdh PE=1 SV=1	GN=Chdh	2.68
181	P29788	Vitronectin OS=Mus musculus GN=Vtn PE=1 SV=2	GN=Vtn	2.70
182	Q9WVC3	Caveolin-2 OS=Mus musculus GN=Cav2 PE=1 SV=1	GN=Cav2	2.71
183	Q60714	Long-chain fatty acid transport protein 1 OS=Mus musculus GN=Slc27a1 PE=1 SV=1	GN=Slc27a1	2.72
184	Q8VCT4	Carboxylesterase 1D OS=Mus musculus GN=Ces1d PE=1 SV=1	GN=Ces1d	2.73
185	Q04857	Collagen alpha-1(VI) chain OS=Mus musculus GN=Col6a1 PE=1 SV=1	GN=Col6a1	2.75
186	P37804	Transgelin OS=Mus musculus GN=Tagln PE=1 SV=3	GN=Tagln	2.78
187	E9QNU1	NAD(P)(+)--arginine ADP-ribosyltransferase OS=Mus musculus GN=Art3 PE=1 SV=1	GN=Art3	2.82
188	Q60994	Adiponectin OS=Mus musculus GN=Adipoq PE=1 SV=2	GN=Adipoq	2.88
189	P47934	Carnitine O-acetyltransferase OS=Mus musculus GN=Crat PE=1 SV=3	GN=Crat	3.00
190	Q91WU0	Carboxylesterase 1F OS=Mus musculus GN=Ces1f PE=1 SV=1	GN=Ces1f	3.01
191	P20239	Zona pellucida sperm-binding protein 2 OS=Mus musculus GN=Zp2 PE=1 SV=1	GN=Zp2	3.01
192	E0CXN5	Glycerol-3-phosphate dehydrogenase [NAD(+)] OS=Mus musculus GN=Gpd1 PE=1 SV=1	GN=Gpd1	3.07
193	Q3UXL1	Protein Akr1cl OS=Mus musculus GN=Akr1cl PE=1 SV=1	GN=Akr1cl	3.08
194	Q05421	Cytochrome P450 2E1 OS=Mus musculus GN=Cyp2e1 PE=1 SV=1	GN=Cyp2e1	3.09
195	Q9DBL9	1-acylglycerol-3-phosphate O-acyltransferase ABHD5 OS=Mus musculus GN=Abhd5 PE=1 SV=1	GN=Abhd5	3.24
196	P15105	Glutamine synthetase OS=Mus musculus GN=Glul PE=1 SV=6	GN=Glul	3.27
197	Q63918	Serum deprivation-response protein OS=Mus musculus GN=Sdpr PE=1 SV=3	GN=Sdpr	3.36
198	P08228	Superoxide dismutase [Cu-Zn] OS=Mus musculus GN=Sod1 PE=1 SV=2	GN=Sod1	3.41
199	P40142	Transketolase OS=Mus musculus GN=Tkt PE=1 SV=1	GN=Tkt	3.43
200	Q91VB8	Alpha globin 1 OS=Mus musculus GN=Hba-a2 PE=1 SV=1	GN=Hba-a2	3.51
201	Q8BH64	EH domain-containing protein 2 OS=Mus musculus GN=Ehd2 PE=1 SV=1	GN=Ehd2	3.51
202	P30115	Glutathione S-transferase A3 OS=Mus musculus GN=Gsta3 PE=1 SV=2	GN=Gsta3	3.52
203	Q3TEA8	Heterochromatin protein 1-binding protein 3 OS=Mus musculus GN=Hp1bp3 PE=1 SV=1	GN=Hp1bp3	3.58
204	D3Z041	Long-chain-fatty-acid--CoA ligase 1 OS=Mus musculus GN=Acsl1 PE=1 SV=1	GN=Acsl1	3.66
205	P51885	Lumican OS=Mus musculus GN=Lum PE=1 SV=2	GN=Lum	4.45
206	O88492	Perilipin-4 OS=Mus musculus GN=Plin4 PE=1 SV=2	GN=Plin4	4.46
207	P49817	Caveolin-1 OS=Mus musculus GN=Cav1 PE=1 SV=1	GN=Cav1	4.92
208	Q08857	Platelet glycoprotein 4 OS=Mus musculus GN=Cd36 PE=1 SV=2	GN=Cd36	4.94
209	P10761	Zona pellucida sperm-binding protein 3 OS=Mus musculus GN=Zp3 PE=1 SV=4	GN=Zp3	5.24

210	P82198	Transforming growth factor-beta-induced protein ig-h3 OS=Mus musculus GN=Tgfbi PE=1 SV=1	GN=Tgfbi	6.12
211	Q02788	Collagen alpha-2(VI) chain OS=Mus musculus GN=Col6a2 PE=1 SV=3	GN=Col6a2	7.21
212	P16015	Carbonic anhydrase 3 OS=Mus musculus GN=Ca3 PE=1 SV=3	GN=Ca3	14.88
213	P04117	Fatty acid-binding protein, adipocyte OS=Mus musculus GN=Fabp4 PE=1 SV=3	GN=Fabp4	14.90
214	F7A0B0	Myelin basic protein (Fragment) OS=Mus musculus GN=Mbp PE=1 SV=1	GN=Mbp	16.62
